# Biomedical Text Link Prediction for Drug Discovery: A Case Study with COVID-19

**DOI:** 10.3390/pharmaceutics13060794

**Published:** 2021-05-26

**Authors:** Kevin McCoy, Sateesh Gudapati, Lawrence He, Elaina Horlander, David Kartchner, Soham Kulkarni, Nidhi Mehra, Jayant Prakash, Helena Thenot, Sri Vivek Vanga, Abigail Wagner, Brandon White, Cassie S. Mitchell

**Affiliations:** 1 Laboratory for Pathology Dynamics, Biomedical Engineering, Georgia Institute of Technology and Emory University, Atlanta, GA 30332, USA; kmccoy8@gatech.edu (K.M.); sgudapati3@gatech.edu (S.G.); lhe80@gatech.edu (L.H.); ehorlander3@gatech.edu (E.H.); david.kartchner@gatech.edu (D.K.); skulkarni302@gatech.edu (S.K.); nmehra3@gatech.edu (N.M.); prakash1.jayant@gmail.com (J.P.); hthenot3@gatech.edu (H.T.); svanga3@gatech.edu (S.V.V.); abbywagner@gatech.edu (A.W.); brandonleewhitejr@gatech.edu (B.W.); 2 Computer Science, Georgia Institute of Technology, Atlanta, GA 30332, USA; 3 Computer Science and Engineering, Georgia Institute of Technology, Atlanta, GA 30332, USA; 4 Biochemistry, Georgia Institute of Technology, Atlanta, GA 30332, USA; 5 Institute for Machine Learning, Georgia Institute of Technology, Atlanta, GA 30332, USA

**Keywords:** COVID-19, SARS-CoV-2, repurposed drugs, coronavirus, natural language processing, text mining, machine learning, literature review

## Abstract

Link prediction in artificial intelligence is used to identify missing links or derive future relationships that can occur in complex networks. A link prediction model was developed using the complex heterogeneous biomedical knowledge graph, SemNet, to predict missing links in biomedical literature for drug discovery. A web application visualized knowledge graph embeddings and link prediction results using TransE, CompleX, and RotatE based methods. The link prediction model achieved up to 0.44 hits@10 on the entity prediction tasks. The recent outbreak of severe acute respiratory syndrome coronavirus 2 (SARS-CoV-2), also known as COVID-19, served as a case study to demonstrate the efficacy of link prediction modeling for drug discovery. The link prediction algorithm guided identification and ranking of repurposed drug candidates for SARS-CoV-2 primarily by text mining biomedical literature from previous coronaviruses, including SARS and middle east respiratory syndrome (MERS). Repurposed drugs included potential primary SARS-CoV-2 treatment, adjunctive therapies, or therapeutics to treat side effects. The link prediction accuracy for nodes ranked highly for SARS coronavirus was 0.875 as calculated by human in the loop validation on existing COVID-19 specific data sets. Drug classes predicted as highly ranked include anti-inflammatory, nucleoside analogs, protease inhibitors, antimalarials, envelope proteins, and glycoproteins. Examples of highly ranked predicted links to SARS-CoV-2: human leukocyte interferon, recombinant interferon-gamma, cyclosporine, antiviral therapy, zidovudine, chloroquine, vaccination, methotrexate, artemisinin, alkaloids, glycyrrhizic acid, quinine, flavonoids, amprenavir, suramin, complement system proteins, fluoroquinolones, bone marrow transplantation, albuterol, ciprofloxacin, quinolone antibacterial agents, and hydroxymethylglutaryl-CoA reductase inhibitors. Approximately 40% of identified drugs were not previously connected to SARS, such as edetic acid or biotin. In summary, link prediction can effectively suggest repurposed drugs for emergent diseases.

## 1. Introduction

Machine learning and text mining tools are used to identify new patterns or links between disparate data. Common applications that predict links in heterogeneous data between concepts of interest include friend suggestions on social media or ad suggestions based on customer segmentation data. However, similar text mining and artificial intelligence tools can be adapted to identify new links in the biomedical literature. Mining of heterogeneous text consisting of concepts such as genes, proteins, diseases, symptoms, and drugs, can expedite predictive medicine, including key predictive tasks such as novel therapeutic discovery or drug repurposing.

The importance of literature based discovery tools for predictive medicine becomes more prominent when new diseases emerge, such as the recent severe acute respiratory coronavirus 2 (SARS-CoV-2, also known as COVID-19) pandemic. COVID-19 is caused by a novel coronavirus, namely SARS-CoV-2, which originated in Wuhan, China. Within a few months after the outbreak, thousands of preprints related to COVID-19 were published [1]. Per the COVID-19 Open research dataset (CORD-19) [2], there were approximately two hundred thousand scholarly articles about COVID-19 and related coronaviruses with 40% of the documents published in 2020. Having readily available text mining tools to quickly analyze large databases of preprints and existing published research articles is important for quickly and efficiently identifying relationships that assist with drug repurposing, risk identification, and mechanisms that could contribute to new therapeutic development.

Modeling the complex heterogeneous data that makes up biomedical literature in the form of a knowledge graph (KG) is the ideal choice [3]. A KG is a network consisting of labeled nodes and edges where nodes represent concepts (genes, proteins, diseases, symptoms, etc.) and edges the relationship(s) between concepts (treats, causes, associates, etc.). Examples: COVID-19 -> *causes* -> pneumonia, remdesevir -> *treats* -> COVID-19, inflammation -> *coexists with* -> COVID-19. A KG has several advantages - it enables end-to-end learning, it can integrate data from multiple sources, and enables continuous integration of heterogeneous knowledge. End to end learning is an artificial intelligence deep learning technique where the model learns all the steps between the initial input phase and the final output result and where all of the different parts of the model are simultaneously trained instead of sequentially trained. End-to-end learning on knowledge graphs can be used for several tasks like link prediction [4], node (or entity) classification [5] and question answering [6]. These methods can be applied to solve several biomedical problems [7] such as drug repurposing, drug discovery, patient diagnosis, drug recommendations, and drug interactions.

Although knowledge graphs are able to model large amounts of data and facts, they still suffer from incompleteness [8] due to missing facts in the literature. Discovering missing facts using existing relationships in the literature is called *link prediction*. Most of the existing link prediction methods learn low dimensional representations of the entities and relations called knowledge graph embeddings. Learned embeddings are then used to infer new relations [4,9,10,11,12]. Link prediction in knowledge graphs has been used for several applications in the biomedical domain. Mohamed et. al. [13] used link prediction for drug target interactions to identify on-target or off-target drugs. Using biomedical knowledge bases such as KEGG [14] or DrugBank [15] a knowledge graph was constructed using entities that connected drugs with their potential targets.

Knowledge graph embeddings and link prediction models have a wide range of applications in the biomedical domain. While several different methods are available, few have integrated extremely large biomedical corpora across multiple domain types into predictions that result in translational, actionable knowledge. In the present work, an end-to-end pipeline was developed that transforms data from various large biomedical corpora into a knowledge graph, learns the embedding representations of entities and relations using knowledge graph embedding based methods, and exposes the results through a web application for real time usage by domain researchers.

The goal of the present case study was to utilize the presented end-to-end link prediction pipeline to identify and rank potential repurposed drugs for COVID-19 using one of the largest and most comprehensive heterogeneous knowledge graphs, SemNet [16], as a foundation. The nearly 30 million PubMed abstracts that comprised SemNet was combined with the CORD-19 data set [2] to create the heterogeneous information network used to identify and rank repurposed drugs for COVID-19. The link prediction tool produced thousands of ranked potential repurposed drugs for the treatment of COVID-19. The benefits of link prediction over existing repurposed drug methodologies is that knowledge is incorporated from all biomedical domains, increasing the likelihood of novel discoveries that might otherwise be overlooked. For example, link prediction can identify repurposed drugs for COVID-19 using relationships patterns comprehensively obtained from all biomedical domains rather than only focusing on drugs utilized or investigated as part of prior coronavirus infections. Moreover, link prediction ranks repurposed drugs from a comprehensive biomedical knowledge graph in a fraction of the time compared to existing manual reviews or traditional empirical simulations to identify repurposed drugs. The constructed automated link prediction tool accuracy was verified using human in the loop validation to assess prediction accuracy. Note that while the presented case study focused on drug repurposing for COVID-19, care was taken to insure the developed knowledge graph and text mining technology is usable for any disease.

The article is structured as follows: Section 2 discusses more formal definitions of knowledge graphs and their related concepts, methods and evaluation metrics. Section 3 gives an outline of the developed end-to-end link prediction pipeline. Section 4 explains the triple extraction process from biomedical corpora, how the knowledge graph is constructed, and provides an analysis of different entities and relations present in the KG. Section 5 discusses model training, experiments and results. Section 6 explains the model deployment procedure using APIs and web applications. Section 7 presents a detailed case study on drug repurposing for COVID-19, where the developed end-to-end system is used to evaluate drugs or substances that can be repurposed to treat SARS-CoV-2. Finally, Section 8 provides the study Conclusions.

## 2. Link Prediction Methods

The present work applies previous link prediction methods to a create a novel end-to-end framework that predicts missing links in a large heterogeneous biomedical knowledge graph. The prediction of missing links is used to identify repurposed drugs that could be suitable for an emergent disease where little to no literature presently exists. Holistic patterns in the knowledge graph that connect disparate domains are used to complete missing links to the emergent disease of interest. The below subsections are meant to provide an overview of the incorporated methods utilized in the presented link prediction application; for details on a specific method, please see the original cited works.

### 2.1. Knowledge Graph Definition

A knowledge graph (KG) can be more formally defined as a collection of factual triples. Each triple will consist of a head entity (*h*), a tail entity (*t*) and the relation (*r*) between them. Here *h*, *t* ∈ E and *r* ∈ R where, E represents set of all entities and R represent set of all relations present in the KG. *(Human coronavirus, interacts, Coronavirus Infections)*, *(Ribavirin, treats, Severe Acute Respiratory Syndrome)* etc. are few such triples that can be present in a biomedical KG.

**Figure 1:**
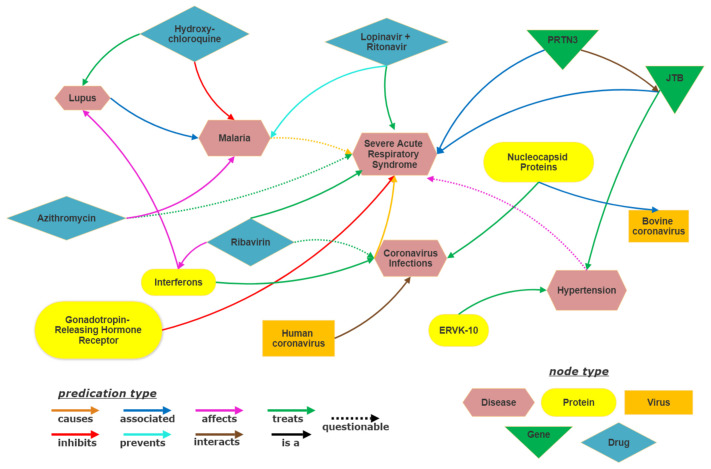
Visualization of subgraph of SemNet Knowledge graph.

The present study utilizes SemNet [16] as the base biomedical knowledge graph (KG). SemNet is a very large, comprehensive semantic inference network that contains relationships extracted from all of PubMed, which is nearly 30 million articles. For the COVID-19 drug repuposing case study, the base SemNet KG is augmented with preprints and new COVID-19 literature [2] as detailed in Section 4. A highly pruned subgraph sampled from the SemNet KG is shown in Figure 1. Although the entire KG is more complex to visualize due to its sheer size, the pruned figure gives a visual representation of the subgraph formed using a few example entities. Along with the entities and relations, the SemNet KG contains information about the types of entities, which are ontological classifications. The entity type is represented by different colors in Figure 1. For example, *Human coronavirus*, *Coronavirus Infections* and *Malaria* belong to the *Disease* entity type.

### 2.2. Link Prediction Task

**Figure 2:**
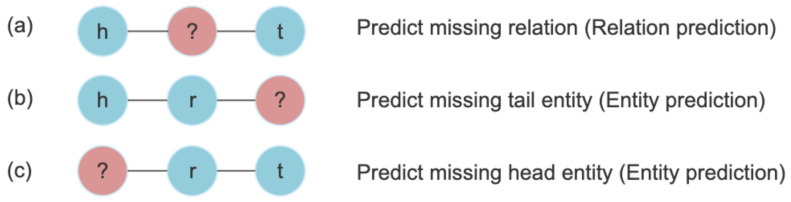
Link prediction and its sub tasks. For a given triple (h,r,t) (**a**) represents Relation prediction task and (**b**,**c**) represent Entity prediction task. Here, h is head entity, t is tail entity and r is relation.

Link prediction or knowledge graph completion is the task of predicting missing relations or missing entities of a triple, as shown in Figure 2. Moreover, prediction of missing relations is referred to as relation prediction and prediction of missing entities is called entity prediction. These tasks can be achieved by several KG embedding methods [8]. Such embedding methods first learn the vector representations for entities and relations. Then, a score function $f(h_{e},r_{e},t_{e})$ is used to measure the salience of a candidate triple (h,r,t). Here, $h_{e},r_{e},t_{e}$ represent embeddings of head entity, relation entity and tail entity, respectively.

Most of these KG embedding methods can be classified into three different families of models, namely: (1) Tensor decomposition models, (2) Geometric models and (3) Deep learning models. Tensor decomposition models consider the KG as a 3D adjacency matrix that can be decomposed into a combination of low dimensional vectors. DistMult, ComplEx and SimplE are examples of tensor decomposition models. Geometric models treat relations as geometric transformations in the latent space. TransE and RotatE are examples of geometric models. As the name suggests, deep learning models use neural networks to perform the link prediction task. Examples of deep neural network models include ConvE [17] and CapsE [18].

In general, any algorithm for learning KG embeddings will have the following steps:

1. Initialize entity and relation embeddings randomly.
2. Generate negative training triples by replacing either the head entity or tail entity of a positive triple with an entity picked randomly from E. Such a process is often referred to as negative sampling.
3. Iterate over the positive and negative triples and update embeddings by optimizing over a loss function that maximizes the score for positive triples and minimizes the score for negative triples. The optimization is completed using a gradient descent algorithm.

The presented algorithm uses the TransE, RotatE and ComplEx methods for KG embeddings training. More details of these methods are described below.

#### 2.2.1. TransE

TransE infers relations as translations in the embedding space. The TransE model was motivated from Word2vec [19], which captures one-to-one relationships between word embeddings as translations. TransE is also effective with hierarchical relationships. It learns embeddings such that the head embedding, when summed with the relation embedding, should fall close to the tail embedding. For a given triple (h,r,t),h+r≈t. TransE uses a distance based scoring function (*f*),

f=−||h+r−t||

#### 2.2.2. RotatE

RotatE defines each relation as a rotation from the head entity to the tail entity in the complex vector space. Similar to TransE, RotatE [12] also belongs to the family of geometric models. The authors [12] demonstrated that RotatE is able to infer several relation patterns like symmetry, anti-symmetry, inversion and composition. For a given triple (h,r,t), RotatE expects t=h∘r. Here, ∘ is the Hadamard or element-wise product. Its corresponding scoring function is

f=−||h∘r−t||

where ∘ indicates the Hadamard product. RotatE introduced a novel self-adversarial negative sampling technique that can be applied to other KG embedding models as well. Instead of uniformly sampling negatives from the data, it uses the current embedding model to sample negative triples.

#### 2.2.3. ComplEx

ComplEx [10] uses latent factorization techniques to learn KG embeddings. It is similar to DistMult model, but uses complex valued embeddings, which allows it to learn anti-symmetry relations in the knowledge graph. The scoring function used is a Hermitian dot product:

f=Re(<h,r,t¯>)

Re(.) indicates the real part of the complex value, <.> indicates the Hermitian product and $\bar{t}$ is the conjugate of t.

### 2.3. Evaluation Metrics

To evaluate these models, two queries (?,r,t) and (h,r,?), were generated for each test triple (h,r,t). Similarly (h,?,t) can also be generated to asses the relationship. For example, if the query is (?,r,t), using every $h^{\prime }\in E$, a score $f(h^{\prime },r,t)$ is calculated using the learned embeddings and the corresponding score function of the model. Results are ranked in the descending order of scores, and model performance is evaluated using the rank of the test triple (h,r,t). The correct items should rank higher than the incorrect items in the ranking order. Moreover, ranking can be performed using a raw or filtered setting. In the filtered setting, the generated triples that already exist in the train or validation sets are removed before computing the ranks.

Mean reciprocal rank (MRR) and hits@k were two popular metrics used for model evaluation. MRR is the harmonic mean of the rank position of the first relevant item. Hits@k represents the proportion of correct items that are predicted in top-k items. The reported results in the present study used filtered MRR and hits@10.

## 3. Link Prediction Pipeline

**Figure 3:**
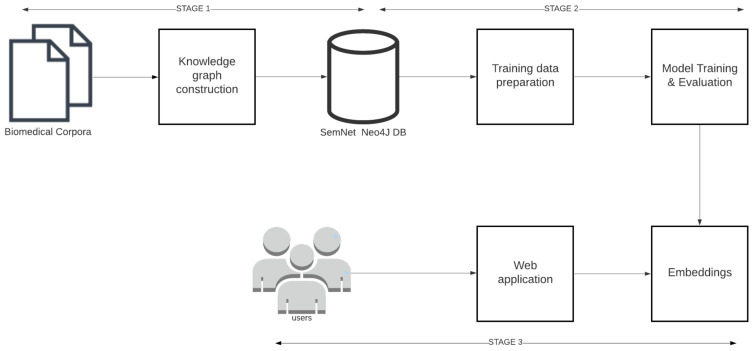
The link prediction pipeline and its 3 main stages: triple extraction, model training, and model deployment.

An end-to-end machine learning pipeline was developed to train and serve link prediction models. As shown in Figure 3, the pipeline consists of three major stages 1. Knowledge graph construction, 2. Model training and 3. Model deployment.

**Knowledge graph construction:** Knowledge graph construction refers to the process of extracting and storing triples from open source biomedical text corpora like PubMed, PubMedCentral, MedArxiv, CORD-19 [2] etc. Knowledge graph construction details are provided in Section 4.

**Model training:** Model training includes the following steps: training data preparation, model training, and implementation to learn KG embeddings. Model training details are provided in Section 5.

**Model deployment:** In model deployment, the trained KG embeddings are deployed through REST APIs and web applications for further assessment by humans as part of case study analyses. Model deployment is explained in Section 6.

## 4. Knowledge Graph Construction

Link prediction is applied to a knowledge graph. The missing links are predicted based on the concepts and relations contained within the knowledge graph. Thus, the selection of the knowledge graph is important. For the present study, a knowledge graph was constructed using SemNet [16], one of the largest and most comprehensive biomedical knowledge graphs available that encompasses nearly all of PubMed, and the CORD-19 dataset [2], which contained newly released and preprint literature on COVID-19.

SemNet [16] is a general-purpose biomedical knowledge graph constructed from 30 million PubMed abstracts and titles. SemNet has 132 ontological types of biomedical concepts and 61 types of semantic predications (i.e., relationship types). Each biological concept is linked to a unique identifier in the Unified Medical Language System (UMLS), a metathesaurus containing over 3.5 million distinct biomedical concepts [20]. This process resulted in approximately 100M semantic predication triples, which were retrieved from SemMedDB [21]. Removing uninformative triples containing one or more generic entities yielded 22M non-generic triples. Concepts with multiple types were consolidated to their most frequent type, ensuring that each UMLS concept represented a single node in the knowledge graph. Since some relationships appeared in multiple abstracts, duplicate triples were merged and assigned edge weights corresponding to the count of how many times each concept appeared among the extracted triples.

**Figure 4:**
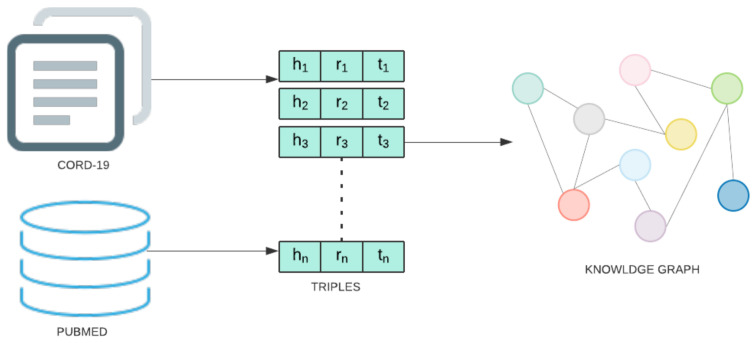
Steps involved in Knowledge graph construction stage.

The knowledge graph utilized for the present study was augmented with COVID-19 information using the CORD-19 dataset [2]. CORD-19 is a collection of research articles assembled by a multilateral government-industry partnership to enable ML-assisted discovery of information that can aid in the treatment, prevention, and understanding of COVID-19. All COVID-19 entity and relation information is extracted from these abstracts and full-text articles using the following pipeline. First, each document is preprocessed by removing sections not amenable to relation extraction (e.g., references) and discarding all documents not written in English. Then, entity and relation information is extracted from SemRep, the same tool used to create SemMedDB. SemRep uses a two-stage pipeline. In the first stage, MetaMap maps textual surface forms to candidate entity and produces a set of linked concepts. Next, SemRep uses a rule-based predication identification module to identify relations between linked entities. This process results in a total of 850,983 extracted relation triples between 74,086 unique entities. A diagram of the information extraction pipeline is given in Figure 4. This data is stored in a Neo4j database.

**Figure 5:**
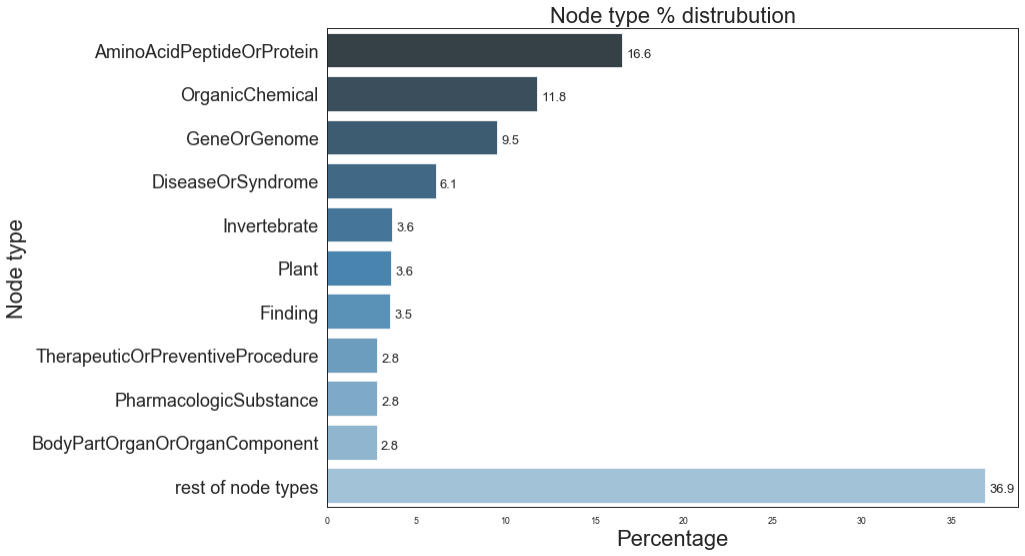
Distribution of most prevalent node types in SemNet ([16]). “Rest of node types” represents the aggregate of remaining node types not individually listed in the figure due to space constraints.

**Figure 6:**
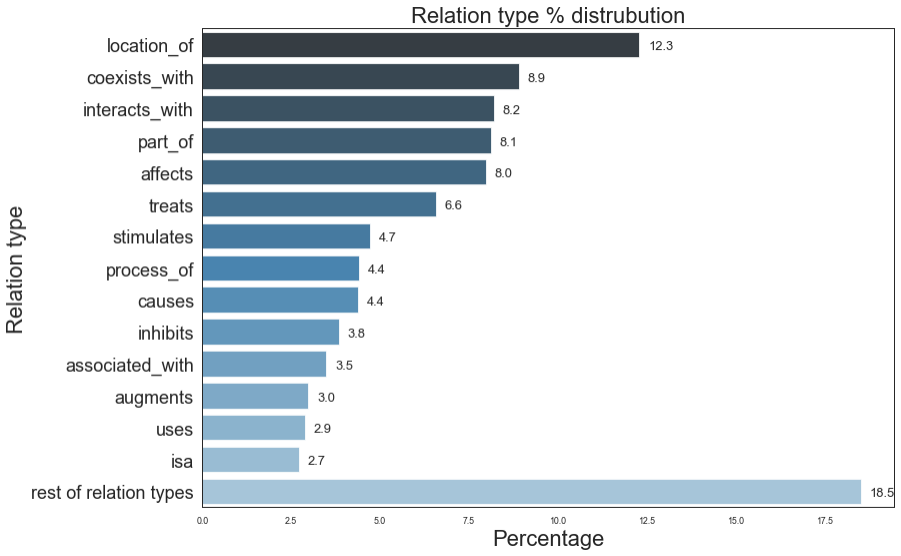
Distribution of different relation types in SemNet ([16]). “Rest of relation types” represents the aggregate of remaining relations types not listed in the figure due to space constraints.

Figure 5 provides information of the most prevalent entity types and their distribution in the Semnet KG. The top 3 major entity types present in KG were AminoAcidPeptideOrProtein (16.6%), OrganicChemical (11.8%), and GeneOrGenomes (9.5%). Figure 6 displays the distribution of different relation types present. The top 3 most frequent relation types are location_of (12.3%), coexists_with (8.9%) and interatcts_with (8.2%).

## 5. Model Training

While the purpose of the presented case study was to identify repurposed drugs for COVID-19, the model was not specifically trained for the aforementioned task. Rather, a more generalizable training approach was utilized as described below to retain the ability to expand future potential applications of the link prediction approach beyond drug repurposing.

### 5.1. Training Data Preparation

Training link prediction models on entire SemNet data yields low evaluation scores, particularly when the domain of interest is a novel, lesser-connected entity, such as COVID-19. Thus, domain specific subgraphs are utilized in link prediction training to improve evaluation scores [22].

As mentioned in the triple extraction section, the CORD-19 dataset is used to collect all COVID related entities. Training data is prepared by extracting all the information related to CORD-19 entities from SemNet. As SemNet has information from both PubMed and CORD-19, extra relations that were not present from CORD-19 are also collected. The data contains information about the head and tail entities, relations, and the type of entities.

While only (h,r,t) triples are used for training, the entity types are abbreviated and appended to the relations. Using the appended form of relations improves evaluation scores of link prediction models when compared with the plain relations. To be precise, the relation *administered_to* is converted to *ADMINISTERED_TO_DIAPadtoHUMN* where *adto* is the short notation of relation; *DIAP* is short form for *DiagnosticProcedure*, which is the head entity type; *HUMN* is short notation for *Human*, which is the tail entity type. The appended form enables the relations to have different contextual meanings based on the head and tail entity types.

After pruning duplicate triples, the data is split into train, validation and test sets. Detailed statistics of the training data are provided in Table 1. The data set contained approximately 9 million triples. However, 50,000 triples were used for validation and test sets. Additionally, the data is converted to a NetworkX [23] graph object, which is used for analysis.

### 5.2. Implementation

Model training is implemented using the DGL-KE library [24] which is a high performance, easy-to-use, and scalable package for learning large-scale knowledge graph embeddings. All the steps related to negative sampling, training, and evaluation of models were carried out using this package. Only the KG embeddings models, namely TransE, CompleX and RotatE, were used for training. Training was done on a server with configuration of 1 NVIDIA TESLA v100 GPU with 32 GB RAM and 48 core CPU with 320 GB RAM. Hyperparameters were fine tuned using the recommended values provided in the library. Final evaluation scores obtained after training were as follows: TransE = 0.40 HITS@10 and MMR = 0.21; ComplEx = 0.44 HITS@10, MMR = 0.25; RotaE = 0.42 HITS@10, MMR = 0.23. Evaluation metrics were computed using the filtered setting.

## 6. Model Deployment

The models are deployed through a set of REST APIs (representational state transfer application programming interfaces) and an easy to use web application. These APIs enable researchers to access the KG embeddings and link prediction results in real time. The web application has three different APIs, which were built using Flask and Python. They are as follows: 1. Embeddings API, 2. Relation prediction API and 3. Entity Prediction API.

### 6.1. Embeddings

**Figure 7:**
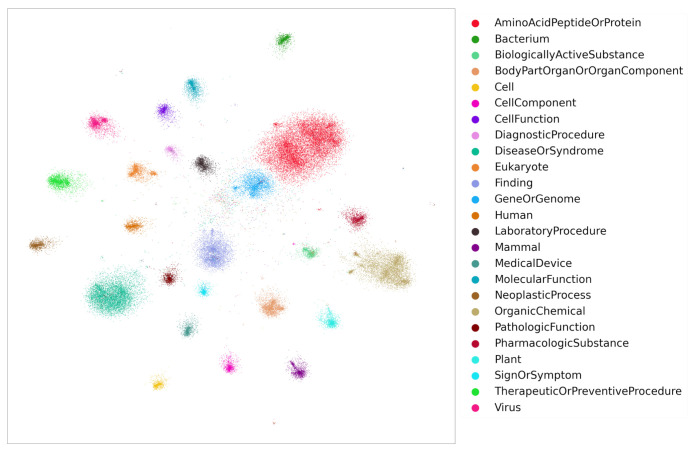
The Entity embeddings (TransE) of top 25 frequent entity groups.

It is well known that word embeddings [19,25,26,27,28] can be used for identifying semantically similar words. After training, the embeddings of similar words fall close to each other in vector space. Likewise, KG entity embeddings bear the same property. To demonstrate, the embeddings obtained from Stage 2 were projected into 2D space using Graphvite [29]. Figure 7 displays the 2D projections of entity embeddings obtained from TransE model training. Several cluster groups are observed where each group represents embeddings of a single entity type. Note that to avoid displaying noisy cluster groups, only the top 25 frequent entity groups were shown in the figure, although the data contains more than 100 entity groups. These embeddings can also be visualized by the open source tool Tensorflow [30] projector. As similar entities fall into one cluster group, the similarity between the entities is measured by distance metrics such as euclidean distance, cosine similarity, etc.

### 6.2. Ranking Predictions

**Figure 8:**
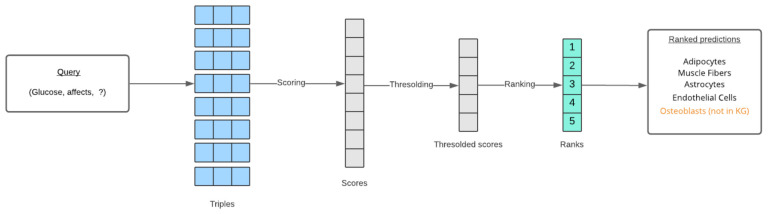
The end-to-end process of ranking link prediction results for a given query.

In both entity prediction and relation prediction tasks, the model predicts a missing entity or relation for a given query (the other two elements of the triple). These predictions are ranked based on a score function specific to each KG model. For example, assume a relation between two entities A and B is to be predicted using the TransE method. A score function for TransE would be used to calculate the scores of all possible triples (A, r, B) formed with every relation present in the data. Scores below a certain threshold are discarded, and the remaining scores are ranked from highest to lowest. These highest ranked relations are returned through the API responses. A similar procedure is followed for entity prediction as demonstrated in Figure 8. Along with the predictions, a boolean value is returned for each prediction, which represents whether or not the triple formed with the prediction is present in the knowledge graph. These new triples are most important to domain researchers. In addition to the TransE, RotatE, and ComplEx methods, an Ensemble method is also added to the APIs. The ensemble method uses a rank aggregation technique [31] to aggregate all the ranked predictions.

### 6.3. REST APIs and User Interface

The REST APIs and user interface are utilized to present link prediction results in a usable form to domain researchers.

#### 6.3.1. Embeddings API

The embeddings API takes arguments of entity *(entity)*, number of neighbors *(size)*, and the model type *(method)*, as inputs. The response contains the specified number of nearest neighboring entities and the corresponding entity types. Nearest neighbors are calculated using the euclidean distance. For example, results obtained from the Embedding API are listed in Table 2. The neighboring entities belong to the same entity group as the query entity. For example, *SARS coronavirus* belongs to the entity type *Virus* and its neighboring entities are from the same group.

The relation prediction API takes arguments of two entities *(head entity, tail entity)* as input and predicts the relation between them. Additionally, number of predictions *(size)* can be specified as an argument. The API response also contains a boolean parameter *(novel)*, which indicates whether the prediction is already present in the KG. Sample results from the API can be found in the Table 3. Although the relations displayed in the table are not in the appended format, in actual usage they are. For example, the top ranked relation between *(Chloroquine, SARS Coronavirus)* is *TREATS_ORCHtrtsVIRS*. Here *ORCH* is the short form for *OrganicChemical* which is the entity type of *Chloroquine*.

#### 6.3.2. Entity Prediction API

The entity prediction API accepts *(entity)* and *(relation)* as arguments and returns the top ranked predictions. Additional input parameters include the boolean parameter *(is_head)* to specify whether input entity is at head or tail of the triple, number of predictions *(size)* and type of the model *(method)*. Similar to the Relation prediction API, it contains a boolean parameter to indicate whether the predictions already exist in the KG. A few example results are shown in Table 4.

#### 6.3.3. User Interface

These APIs are deployed through a web application. Its user interface (UI) is built using RESTool [32], which is an open source UI tool to manage REST APIs. By entering arguments *(Entity: Virus, size: 5, Method: Complex)*, the top 5 nearest entities to Virus were retrieved. Similar UI pages exist for Entity and Relation prediction APIs as well.

### 6.4. Locality Sensitive Hashing

One of the goals of the web app design is to provide low latency response to the end user. All the APIs described above require nearest neighbor computations. Nearest neighbor computations cannot be performed “on the fly” as it would require a linear scan through the entire dataset. When the dataset size is large as in this case, even a linear scan takes a large amount of time. Locality sensitive hash functions are specifically designed so that hash value collisions are more likely for two data points that are closer together than for data points that are far apart. This property is exploited by locality sensitive hashing [33] to avoid slow linear scans while finding nearest neighbors. The APIs use Annoy [34] which is a publicly available locality sensitive hashing library.

## 7. COVID-19 Case Study

Due to its current relevance, COVID-19 was chosen as the application upon which to utilize and validate the developed end-to-end pipeline for literature link prediction. The goal of the present case study was to utilize the presented end-to-end link prediction pipeline to identify and rank potential repurposed drugs for COVID-19.

### 7.1. Case Study Background

The novel coronavirus, officially named SARS-CoV-2, is named for the disease it causes in infected hosts, Severe Acute Respiratory Syndrome (SARS). SARS-CoV-2 differs from its other coronavirus predecessors in that it is more highly infectious [35], which resulted in a global pandemic. SARS-CoV-2 is also known to cause respiratory distress that is particularly dangerous to the elderly and those with common chronic diseases, including cardiovascular disease, chronic obstructive pulmonary disease, diabetes mellitus, and others. SARS-CoV-2 ignited an urgent search for therapeutic measures, which may be used to improve prognosis for critical patients. Repurposed medications, which have already been approved for use by the United Stated Food and Drug Administration (FDA) or similar approval bodies in other countries, have shown promise in treating SARS-CoV-2. Repurposed drugs, which have previously undergone prior safety testing, are the fastest means to initiate treatment until specific etiology-dependent drugs can be developed and tested. Clinical trials continue for a multitude of treatments ranging from antiviral medications to antimalarials, contributing to a growing body of SARS-CoV-2 research.

The goal of the present case study was to utilize the presented end-to-end link prediction pipeline to identify and rank potential repurposed drugs for COVID-19. Predictions are based on the presented augmented knowledge graph, which contained information on previous SARS infections, such as the 2002 SARS coronavirus and the 2012 Middle East respiratory syndrome (MERS) coronavirus, along with early released COVID-19 articles from the CORD data set.

Using link prediction, meaningful relationships between SARS-Cov-2 and potential treatments and/or techniques for disease management were identified. Subsequently, relationships were validated using human in the loop inspection of full text literature to confirm, if possible, the relationship and its potential clinical therapeutic context. Finally, validated relationships were compiled to produce a list of predicted drugs and procedures that are most relevant to SARS-CoV-2. The outcome was twofold: (1) The prioritized therapeutics lists for SARS-CoV-2 provide important insight to effectively expedite further clinical translation; (2) the link prediction pipeline was validated, providing evidence that it can not only be used successfully for drug repurposing for COVID-19 but could also be adapted and trained to make predictions for other diseases or use cases.

### 7.2. Case Study Methods

The developed end-to-end pipeline for link prediction was used in combination with literature based discovery tool, SemNet, as described in Section 4, Section 5 and Section 6, to identify and rank therapeutic drugs and procedures that were predicted to be important to SARS-CoV-2. The below methods for the COVID-19 case study focus on the human in the loop validation and quality control to affirm the utility, accuracy, and context of the link prediction tool node rankings.

#### 7.2.1. Link Prediction Validation Label Assignment

Human in the loop review of full text literature was essential to validate the developed link prediction tool and to provide important context for its highly ranked nodes. The lowest 20 HeteSim ranked concepts (lower HeteSim scores indicate closer relationship) of 9 drug classes (180 of 2852 total nodes) were selected and used as keyword searches for literature relating each concept to SARS coronavirus (SARS-CoV) and COVID-19 (SARS-CoV-2). The validation labels PROVEN, DISPROVEN, UNCLEAR, and MISSING were assigned to each predicted link during the human in the loop validation process with the purpose of corroborating or rejecting the results of the link prediction tool by reviewing full text COVID-19 literature. Literature review to validate the predicted COVID-19 links from the algorithm was conducted in three steps. First, researchers conducted literature searches in PubMed using the following format: “*insert_node_name* SARS coronavirus”. Next, the results of these literature searches were reviewed by 5 different trained human in the loop validators. Finally, a quality control leader reviewed the labels assigned by each validator for each search to ensure validation integrity adherence to strict labelling protocols. The importance of the human in the loop validation is that humans can gauge context in ways that algorithms cannot. Finally, human in the loop validation provided a ground truth by which to assess the performance of the developed link prediction algorithm.

The label PROVEN was assigned when an article detailed the repurposed drug having the same relationship to SARS-CoV or SARS-CoV-2 as the link prediction tool, in vitro or in vivo. For instance, the antimalarial *Methotrexate* was listed as having a *treats* relationship as its first predicted link. Therefore, human validators assigned to the antimalarials drug class would subsequently complete a search in PubMed with the format “Methotrexate SARS coronavirus” in order to find full text articles that contain contextual information on the relationship. Although the search was conducted with the keyword SARS coronavirus, any articles involving the treatment (Methotrexate in this case) and SARS-CoV-2 would be sufficient since the two viruses are considered to be closely related. It was found that Methotrexate inhibits the replication of SARS-CoV-2 in model cell lines, thus “proving” that *Methotrexate treats* SARS-CoV-2 in vitro [36]. Therefore, the relationship *treats* was considered PROVEN.

The label DISPROVEN was assigned when the predicted link was proven to be incorrect, or in cases where the opposite instance occurred. For example, the node *polyamines* was predicted to *treat* SARS coronavirus. In a full text COVID-19 study, it was found that *polyamines* are molecules that are synthesized within human cells and promote the replication of SARS-CoV-2 [37]. Thus, the relationship *treats* was considered DISPROVEN.

The label UNCLEAR was assigned when the available full text COVID-19 literature regarding the true relationship was ambiguous. UNCLEAR was also used in cases where a drug had been suggested for treatment of SARS coronavirus, but no actual studies have been conducted. For example, it was stated that the first predicted link for the anti-inflammatory *ebselen* is *treats*. Human in the loop validation on this node revealed that there is potential for inhibition of the main protease of SARS-CoV-2. In order to deduce this, multiple in silico analyses were run [38]. These findings did not indicate that *ebselen* was able to inhibit the replication of SARS-CoV-2 in vitro or in vivo, meaning the relationship would be labelled as UNCLEAR.

A label of MISSING was assigned when no full text COVID-19 articles deducing the true relationship were present at the time of evaluation. The protease inhibitor *tretinoin* was seen to have a primary predicted link of *treats*. However, there were no articles relating it to SARS-CoV or SARS-CoV-2. As a result, the relationship was deemed to be MISSING.

#### 7.2.2. Pharmacokinetic Label Assignment

Pharmacokinetic labels were assigned by human in the loop validators when the validation label was either PROVEN or DISPROVEN for SARS-CoV-2. Note that these human-assigned labels in no way impacted the link prediction rankings. Rather, pharmacokinetic labels only provide additional context when interpreting the predicted therapeutic substances the algorithm deemed relevant to SARS-CoV-2. Therapeutic substances were divided into four broad pharmacokinetic categories: ‘primary’ therapies, ‘adjunctive’ therapies, ‘side-effect’ therapies, and ‘other’.

As part of the labelling protocol, ‘primary’ therapies were defined as treatments that combat the viral infection, itself, and prevent or limit its replication within the body. *Methotrexate* was mentioned above as an antimalarial treatment that was confirmed to have a PROVEN relationship of *treats* with regard to SARS-CoV-2. *Methotrexate* works to inhibit the replication of SARS-CoV-2, and thus was labeled as a ‘primary’ therapy.

‘Adjunctive’ therapies were defined as secondary treatments, which enhance the antiviral effects of primary therapies when delivered in combination. *Albuterol*, a member of the anti-inflammatory drug class, was confirmed by validators to have a PROVEN relationship of *treats* SARS-CoV-2. In the validation article(s), *albuterol* was administered in combination with nebulized dornase alfa to improve the delivery of this therapy to the lungs in patients infected with SARS-CoV-2 [39]. Therefore, a pharmacokinetic label of ‘adjunctive’ was assigned to this treatment.

‘Side-effect’ therapies were defined as treatments or medications, which do not directly target the virus, but instead improve patient prognosis by attenuating the severity of inflammation, immune agitation, dyspnea, dysphagia, pneumonia, or other symptoms associated with coronavirus infection. *Ciprofloxacin* is listed in both the immunomodulators and antimalarials drug classes and possesses a PROVEN relationship of *treats* SARS-CoV-2. The pharmacokinetic label for this treatment was deemed ‘side-effect’. For example, *Ciprofloxacin* was used to reduce the increased effects of pneumatosis intestinalis in a patient due to SARS-CoV-2 [40].

If the pharmacokinetic labels, as defined by the study protocol methods above, did not apply to a target node, a label of ‘other’ was assigned. ‘Other’ was synonymous to a miscellaneous category for nodes that have either different and/or mixed relationships that do not fit within the primary, adjuvant, or side effect labels. The node *Nucleocapsid* refers to nucleocapsid proteins, which possesses a PROVEN relationship of *part of* SARS-CoV, meaning it is a component of the virus. During human in the loop validation, it was observed that SARS-CoV contains a nucleocapsid protein that binds to DNA in vitro as well as interferes with cellular processes in hosts [41]. Considering its mixed effects, *Nucleocapsid* was given a pharmacokinetic label of ‘other’.

### 7.3. Case Study Results

#### 7.3.1. HeteSim Score Distributions for Link Prediction Validation Labels

A validation label was assigned (PROVEN, DISPROVEN, UNCLEAR, or MISSING) to the top 20 source nodes in each drug class to evaluate the accuracy of the predictions made by the link prediction tool. The top 5 results, ranked by lowest HeteSim scores, for each assignment are shown in Table 5, respectively. The complete list of top ranked nodes (n = 180, equivalent to the top 20 ranked nodes for each of the most prevalent 9 node types shown in Figure 5) can be found in Supplementary Table S1. Supplementary Table S1 illustrates the node, node type, SemNet feature scores (HeteSim score, standardized HeteSim score, etc.) the link prediction algorithm’s predicted link(s), human in the loop validation labels and corresponding full text validation evidence, and the study-assigned pharmacokinetic labels.

There is a very strong correlation between the number of source nodes in a drug class and the median HeteSim score of the source nodes in that drug class (Kendall tau rank correlation, τ=0.944). Thus, it is difficult to directly compare the HeteSim score distributions of different drug classes or to directly compare the HeteSim scores of source nodes in different classes. To further lessen Count correlation for the present SARS-CoV-2 study, a standardized HeteSim score was defined to divide the HeteSim scores of the source nodes in each class by the number of nodes in that particular class.

**Figure 9:**
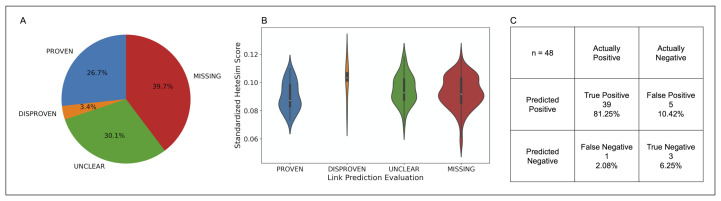
(**A**) Pie chart illustrating the composition of the COVID-19 case study dataset by link prediction evaluation. (**B**) Violin plot showing the distribution of standardized HeteSim scores between each link prediction evaluation. Lower HeteSim means a closer relationship between the source node and tail node. (**C**) Confusion matrix for the link prediction in the COVID case study. “MISSING” and “UNCLEAR” nodes were left out as the true relationship is unknown. Sensitivity = 0.975, specificity = 0.375.

After standardization, the nodes in the PROVEN category were found to have the lowest median standardized HeteSim score, followed in order by MISSING, UNCLEAR, DISPROVEN. Based on a Kruskal-Wallis test, these four categories of source nodes are significantly different in the distribution of their standardized HeteSim scores (p=0.041). Upon performing the pairwise Mann-Whitney tests with a Bonferroni correction, it was discovered that no two pairs of link prediction validation labels are significantly different in their standardized HeteSim score distribution. The makeup, distribution, and analysis of the relationship evaluations is shown in Figure 9.

The link-prediction accuracy (LPA) is a measure to assess how accurate the simulation’s links are in comparison to human in the loop validation, where human in the loop validation served as the ground truth. LPA was calculated by dividing the number of PROVEN links by the total number of DISPROVEN and PROVEN links. The LPA for the top 20 nodes of all the drug classes is 0.875. Furthermore, the sensitivity and specificity of the link prediction tool was calculated with results as shown in part C of Figure 9.

The embeddings API takes arguments of entity *(entity)*, number of neighbors *(size)*, and the model type *(method)* as inputs. The response contains the specified number of nearest neighboring entities and the corresponding entity types. Nearest neighbors are calculated using the euclidean distance. For example, results obtained from the Embedding API are listed in Table 2. It can be observed that the neighboring entities belong to the same entity group as the query entity. For example, *Tobacco* belongs to entity type *HazardousOrPoisonousSubstance* and its neighboring entities also are from the same group.

#### 7.3.2. HeteSim Score Distributions for Pharmacokinetic Labels

Pharmacokinetic categories were assigned to each of the drugs used to treat patients with SARS-CoV-2 or SARS-CoV.

**Figure 10:**
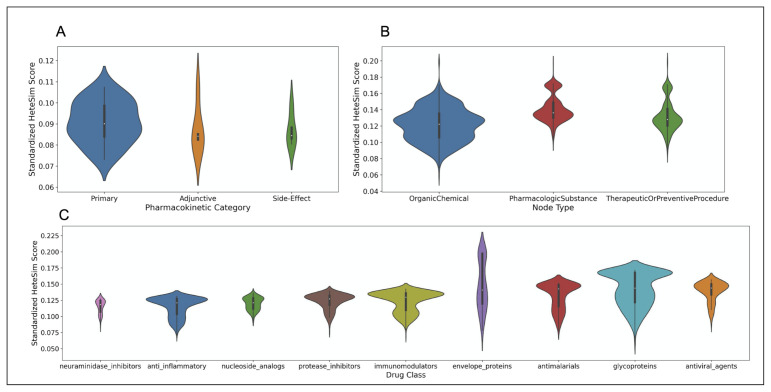
(**A**) Violin plot showing the distribution of standardized HeteSim scores between each pharmacokinetic label. Lower HeteSim score means a closer relationship between the source node and tail node. (**B**) Violin plot showing the distribution of standardized HeteSim scores between each node type. (**C**) Violin plot showing the distribution of standardized HeteSim scores between each drug class.

After standardization, the nodes in the ‘primary’ category have the lowest standardized HeteSim score, followed in order by ‘side-effect’, ‘adjunctive’, and ‘other’. Based on a Kruskal-Wallis test, there is no significant difference between the standardized HeteSim score distributions of the drugs labeled ‘primary’, ‘side-effect’, and ‘adjunctive’ (p=0.51). A violin plot illustrating the various distributions of standardized HeteSim scores is shown in part A of Figure 10.

#### 7.3.3. HeteSim Score Distributions for Different Drug Classes

After standardizing the HeteSim scores, the drug classes of *neuraminidase inhibitors*, *anti-inflammatory*, and *nucleoside analogs* have the lowest median standardized HeteSim scores, in ascending order. The Kruskal-Wallis test finds that the distributions of the standardized HeteSim scores of the drug classes are significantly different $\left(p=2.80*10^{-129}\right)$. Based on pairwise Mann-Whitney tests with the Bonferroni correction, the standardized HeteSim score distribution for each pair of drug classes is significantly different with a few exceptions. These exceptions are *anti-inflammatory* & *neuraminidase inhibitors* (p=1), *anti-inflammatory* & *nucleoside analogs* (p=0.57), *antimalarials* & *envelope proteins* (p=0.21), *antiviral agents* & *glycoproteins* (p=0.21), *immunomodulators* & *protease inhibitors* (p=0.23), and *neuraminidase inhibitors* & *nucleoside analogs* (p=0.058). A violin plot illustrating the various distributions of standardized HeteSim scores is shown in part C of Figure 10.

#### 7.3.4. HeteSim Score Distributions for Different Node Types

The node types returned in the SemNet simulations included *OrganicChemical*, *TherapeuticOrPreventiveProcedure*, and *PharmacologicSubstance*, as well as *AminoAcidOrProtein*, *Receptor*, *ImmunologicFactor*, *GeneOrGenome*, and *BiomedicalOccupationOrDiscipline*. The latter 5 node types were deemed irrelevant to the repurposed drugs for SARS coronavirus, and only comprised 103 of the total 2852 nodes. Therefore, they are excluded from the analysis. After standardization, *OrganicChemical* nodes had the lowest median standardized HeteSim scores, followed by *TherapeuticOrPreventiveProcedure*, and then *PharmacologicSubstance*. Based on a Kruskal-Wallis test, the standardized HeteSim distribution of the three node types are significantly different in the distribution of their standardized HeteSim scores $\left(p=7.60*10^{-97}\right)$. Based on pairwise Mann-Whitney tests with a Bonferroni correction, each pair of node types has significantly different standardized HeteSim scores. A violin plot illustrating the various distributions of standardized HeteSim scores is shown in part B of Figure 10.

### 7.4. COVID-19 Results Interpretation and Discussion

The top 20 nodes in each predominant node type (n = 180 nodes, see Supplementary Table S1 for complete information) were assigned a link prediction validation label: PROVEN, DISPROVEN, UNCLEAR, or MISSING. Numerous primary therapies, mainly repurposed drugs, were identified with the potential to counteract disease progression. Proven primary treatments included *human leukocyte interferon*, *recombinant interferon-gamma*, *cyclosporine*, *antiviral therapy*, *zidovudine*, *chloroquine*, *vaccination*, *methotrexate*, *artemisinin*, *alkaloids*, *glycyrrhizic acid*, *quinine*, *flavonoids*, *amprenavir* and *suramin*. Proven adjunctive treatments included *complement system proteins*, *fluoroquinolones*, *bone marrow transplantation*, and *albuterol*. Proven drugs for side effects were *ciprofloxacin*, *quinolone antibacterial agents*, and *hydroxymethylglutaryl-CoA reductase inhibitors*.

#### 7.4.1. Value of Link Prediction for Emergent Diseases

Through the use of semi-supervised machine learning models to mine millions of academic journal articles, relationships between repurposed therapeutics and SARS coronavirus can be predicted with link prediction. SARS-CoV and SARS-CoV-2 possess many similarities structurally, which can assist in predicting SARS-CoV-2 even before there is substantial SARS-CoV-2 specific data [42]. For the sake of discovering therapies in the absence of little to no data for a specific emergent disease, link prediction is a valid starting point for drug repurposing. For example, in the present SARS-CoV-2 case study, nodes where specific human in the loop validation of evidence for efficacy is MISSING are potential novel therapeutics that should be prioritized for future experimental in clinical assessment for SARS-CoV-2. Essentially, the human in the loop validation and link prediction accuracy of 0.875 illustrate that link prediction is able to legitimately predict valid repurposed drugs for emergent diseases even when the model has relatively little to no access to literature specific to the emergent disease.

#### 7.4.2. HeteSim Score Distributions for Labels assigned through Human in the Loop Validation

HeteSim score distributions for drugs labeled PROVEN, DISPROVEN, UNCLEAR, and MISSING are significantly different and biased towards PROVEN, MISSING, and UNCLEAR nodes. A logical interpretation of this finding is SemNet is uncovering new relationships among nodes that are not explicitly stated or explained in literature. For instance, if SemNet simply regurgitated the findings of papers in PubMed, the HeteSim scores of the PROVEN category would be significantly lower than the HeteSim scores of the UNCLEAR category. However, the HeteSim scores in the PROVEN and UNCLEAR categories are not significantly different, which illustrates that the SemNet ranking algorithm may be exploring connections between repurposed drugs and SARS that have not been confirmed by clinical trials. This hypothesis warrants further testing, which can help improve the trustworthiness and generalizability of SemNet for guiding drug discovery.

#### 7.4.3. HeteSim Score Distributions for Pharmacokinetic Labels

The HeteSim score distributions for pharmacokinetic labels are the only comparison that was not found to be significantly different. The main interpretation from this analysis is that each of the pharmacokinetic labels are equally connected and ranked similarly. ‘Primary’ therapies were the most represented, likely due to this study’s focus on repurposed drugs with the potential to counteract disease progression. The top ranked ‘primary’ treatment is *human leukocyte interferon*, a signaling protein that has been shown to inhibit SARS replication with high selectivity [43]. The ‘adjunctive’ and ‘side-effect’ therapies resulted in fewer validated treatments than the ‘primary’ therapies. This was in large part due to the difficulty in finding ground truth verification data for the link predictions in the relatively new, fewer, and lesser connected COVID-19 specific publications. The top ranked ‘adjunctive’ treatment is *Fluoroquinolones*, a class of antibiotics found to be effective adjuvants to other SARS treatments. The top ranked ‘side-effect’ treatment is *Ciprofloxacin*, a fluoroquinolone antibiotic used to reduce the increased effects of pneumatosis intestinalis in patients with SARS-CoV-2 [40].

#### 7.4.4. HeteSim Score Distributions for Different Drug Classes

The ranking of the drug classes by median standardized HeteSim score is generally corroborated by available COVID-19 specific evidence. *Anti-inflammatory* drugs, the second-ranked class by this metric, are used to treat patients with SARS-CoV-2; inflammation caused by immune system overreaction is a common phenomenon in SARS-CoV-2 patients. *Nucleoside analogs*, the third-ranked class, include *remdesivir*; the efficacy of *remdesivir* has been widely studied in clinical trials with patients suffering from SARS-CoV-2 [44]. Interestingly, *protease inhibitors* and *antimalarials* were of middle ranking importance. *Protease inhibitors*, including *lopinavir* and *ritonavir*, have been studied extensively as potentially effective repurposed drugs [45]. *Antimalarials* such as *Chloroquine* have also been studied as potentially effective repurposed drugs [46]. The *Envelope proteins* class and the *Glycoproteins* class ranked more poorly based on median HeteSim score, which is unsurprising since these two classes do not describe drugs, but instead, describe components of viruses [47,48]. More favorably ranked classes have lower median HeteSim scores. The SemNet algorithm found that such classes have more promising connections with SARS, and the drugs in these higher ranked (i.e., more favorable) classes may yield better efficacy in treating COVID-19 patients in future clinical trials.

#### 7.4.5. HeteSim Score Distributions for Different Node Types

*OrganicChemical* nodes have the lowest median standardized HeteSim scores, followed by *TherapeuticOrPreventiveProcedure*, and then *PharmacologicSubstance*. *OrganicChemical* nodes include 1558 out of 2852 total nodes. During literature review, *OrganicChemical* nodes were found to be much more relevant than nodes of other types. For example, the top ranked *OrganicChemical* node is *Chloroquine*, but the top *PharmacologicSubstance* and *TherapeuticOrPreventiveProcedure* nodes are *Nucleoside Analogs* and *Antiviral Therapy*, respectively. Because *OrganicChemical* nodes have significantly lower HeteSim scores and comprise most of the nodes returned in the SemNet simulations, it would be wise to focus on only *OrganicChemical* nodes in future work.

### 7.5. Comparison to Existing Drug Repurposing Efforts

In the advent of the COVID-19 pandemic, there has been an unprecedented flood of governmental and scientific support for the search of repurposed drugs. However, most existing scientific reviews of repurposed drugs for COVID-19 center around the same list of a few overexposed drugs [49,50], which are more obvious choices given the structure and history of coronaviruses. In contrast, the text mining based link prediction model developed and utilized in the present study is able to discern less biased patterns beyond the appeal of highly publicized drugs to identify seemingly hidden yet still significant drugs. Furthermore, the developed end-to-end pipeline can rapidly ingest and incorporate new literature and re-rank drugs as new information becomes available. It would be impossible for even a large team of human domain experts to review and iterate at an equivalent volume and speed.

Another repurposed drug tool is molecular modeling. Kandeel et al. [51] used molecular modeling and virtual screening to test for similarities in structure that could be relevant to treating COVID-19. However, this work was limited to only drugs that were once used for previous SARS epidemics. The authors test and compare the compatibility of drugs to the surfaces of SARS-CoV-2 and other coronaviruses. Drugs that have worked for previous coronaviruses and work on similar surface features to SARS-CoV-2 are recommended for further research.

The artificial intellgience (AI) text mining model presented in this work has two main advantages compared to the manual domain expert review and molecular modeling approaches: comprehensiveness and the ability to simulate in real-time. SemNet’s knowledge graph and link prediction work over the entire knowledge base of medicine, and do not narrow the scope of search.

Admittedly, many of the suggested repurposed drugs highlighted by link prediction have already been recommended for further research. However, 39.7% of the suggested repurposed drugs in Supplementary Table S1 have no known or “obvious” relationships to SARS-CoV in literature. It is these drugs that are the most interesting, as they have ranked nearly as well as drugs positively connected to SARS coronavirus in literature, but have yet to be suggested for repurposing for COVID-19. Some examples of drugs or substances with no current connection to SARS coronavirus include edetic acid, biotin, fluoroquinolones, ethyl pyruvate, tretinoin, fucoidan, sulfhydryl compounds, pentetic acid, and sulfonamides. As an example, edetic acid is a chelating agent with anti-hypercalcemic and anticoagulant properties [52]. It readily binds to calcium and heavy metal ions, and is used as an anticoagulant as well as a treatment for heavy metal poisoning. Its relationship to SARS coronavirus is not known, yet the link prediction model highlighted it as a promising repurposed drug for SARS-CoV-2. Metal ions have an interesting, but still unexplored relationship to SARS-CoV-2 and its main protease [53]. Edetic acid could possibly work in a similar manner and disable the main protease of SARS-CoV-2, preventing the fusion of SARS-CoV-2 to the ACE2 receptor, and thereby, prevent or lessen infection. Biotin is another name for Vitamin B7. It is known to play an important role as a co-factor in the regulation of inflammation and transcriptional factors for inflammatory processes [54]. Biotin could possibly interfere with the cytokine storm of SARS-CoV-2 and lessen the symptoms of patients. Fluoroquinolones are a class of antibacterials that work by halting the DNA replication machinery of bacteria [55]. While they are known to treat secondary bacterial infection in COVID-19, link prediction suggests an additional mechanism. Perhaps, this class of compounds could interfere with the RNA replication machinery of SARS-CoV-2, thus decreasing the SARS-CoV-2 viral load in patients. In summary, similar logic can be applied to the other drugs identified as highly ranked by link prediction but where there is no current connection to SARS coronavirus. Likewise, this conclusion applies all the link prediction results with the human in the loop validation label of MISSING.

## 8. Conclusions

The results of the COVID-19 case study using text mining link prediction illustrate that the developed end-to-end link prediction tool provides an effective platform to identify and prioritize repurposed drugs for emergent diseases like SARS coronavirus. Human in the loop validation verified the accuracy of link prediction to be 0.875. Several of the top ranking COVID-19 repurposed drug candidates were previously known to be associated with SARS or have already been tested in COVID-19. However, nearly 40 percent of high ranking COVID-19 repurposed drug candidates predicted by link prediction have not been previously identified by other drug repurposing methods, including molecular structure modeling. The comprehensiveness of the biomedical literature included in link prediction (over 30 million articles) provides breadth not obtainable with other drug repurposing methods. While text mining with link prediction does not replace traditional drug repurposing methods, it can be an important tool to quickly and efficiently increase the breadth of searches for potential repurposed drug candidates in emergent or rare diseases.

## Figures and Tables

**Table 1:** Statistics of the SemNet-COVID training data.

Properties	Statistics
Entities	74,086
Triples	8,928,797
Entity types	121
Relation types	61
Appended relation types	25,341
Training triples	8,828,797
Validation triples	50,000
Test triples	50,000

**Table 2:** Example results from the Embedding API with different input queries. Input Parameters are (entity: as shown in table, size: 5 and method: Complex).

Query: SARS Coronavirus	Query: Chloroquine	Query: Cyclosporine
Middle East Respiratory Syndrome Coronavirus	Hydroxychloroquine	Calcineurin
Genus: Coronavirus	Polymyxin B Sulfate	rituximab
SARS coronavirus Urbani	Aminoquinoline	infliximab
Beluga Whale coronavirus SW1	Bauxite	Calcitonin Receptor
SARS-related bat coronavirus	Mefloquine	Neoral

**Table 3:** Example results from the Relation prediction API with 5 different queries.

Query	Top Ranked Relation
(Chloroquine, ?, SARS Coronavirus)	treats
(SARS Coronavirus, ?, Chloroquine)	location_of
(Dexamethasone, ?, SARS Coronavirus)	treats
(Albuterol, ?, Dornase Alfa)	uses
(Nucelocapsid, ?, SARS Coronavirus)	part_of

**Table 4:** Example results from the Entity prediction API with two different queries. In the first column, the query to the API (entity: SARS coronavirus, relation: treats, size: 5, method: Ensemble, is_head: False) and head entity predictions are displayed. In the second column, query (entity: Chloroquine, relation: Chloroquine, size: 5, method: Ensemble, is_head: True) and the predictions of tail entity are displayed.

Query: (?, treats, SARS Coronavirus)	Query: (Chloroquine, Chloroquine, ?)
Chloroquine	Ebola Virus
Duration	Virus
Glycyrrhizic Acid	SARS coronavirus
Ritonavir	Zika Virus
Octanoic acid	HIV

**Table 5:** The top 5 nodes with each link prediction validation label, ranked by lowest HeteSim scores. Lower standardized HeteSim scores corresponds to a stronger relationship. The complete list of top ranked nodes (n = 180, equivalent to the top 20 ranked nodes for each node type) can be found in Supplementary Table S1.

Node	Drug Class	Node Type	Standardized HeteSim Score	Predicted Link	Pharmacokinetics
Top 5 PROVEN Nodes					
Chloroquine	glycoproteins big	OrganicChemical	0.073	treats	Primary
Glycyrrhizic Acid	anti-inflammatory	OrganicChemical	0.074	treats	Primary
Quinine	anti-inflammatory	OrganicChemical	0.077	treats	Primary
Chloroquine	antimalarial	OrganicChemical	0.077	treats	Primary
Fluoroquinolones	antimalarial	OrganicChemical	0.077	treats	Adjunctive
Top 5 DISPROVEN Nodes					
Polyamines	antimalarial	OrganicChemical	0.080	treats	Other
Complement System Proteins	neuraminidase inhibitors	ImmunologicFactor	0.101	prevents	Other
Dopamine Receptor	neuraminidase inhibitors	Receptor	0.103	treats	Other
Chemokine (C-C Motif) Receptor 5|CCR5	envelope protein	Receptor	0.104	treats	Other
Antiviral prophylaxis	nucleoside analogs	TherapeuticOr PreventiveProcedure	0.107	neg treats	Primary
Top 5 UNCLEAR Nodes					
small molecule	immunomodulators	OrganicChemical	0.069	prevents	N/A
ebselen	anti-inflammatory	OrganicChemical	0.077	treats	N/A
Fluticasone propionate	anti-inflammatory	OrganicChemical	0.079	treats	N/A
Quinolone Antibacterial Agents	anti-inflammatory	OrganicChemical	0.082	prevents	N/A
Morphine	anti-inflammatory	OrganicChemical	0.084	treats	N/A
Top 5 MISSING Nodes					
small molecule	glycoproteins big	OrganicChemical	0.056	prevents	N/A
RABBIT SERUM	glycoproteins big	OrganicChemical	0.061	N/A	N/A
Esters	anti-inflammatory	OrganicChemical	0.070	N/A	N/A
Edetic Acid	glycoproteins big	OrganicChemical	0.074	treats	N/A
small molecule	protease inhibitors	OrganicChemical	0.075	N/A	N/A

## Data Availability

SemNet software and Link Prediction code is available at GitHub, www.github.com/pathology-dynamics, or by contacting the corresponding author.

## Supplementary Materials

The following are available online at https://www.mdpi.com/article/10.3390/pharmaceutics13060794/s1, Supplementary Table S1: Top Ranked Link Prediction Nodes for Potential COVID-19 Treatment. Predicted nodes are primarily based on analyzing connections in the knowledge graph from older coronavirus literature on SARS and MERS. Lower hetesim score is better. Standardize hetesim score used for comparison. Predicted link shows predicted relationship type. DOI, validation label, pharmacokinetic label determined by human in the loop validation using multiple trained human reviewers with quality control examining existing COVID-19 specific data sets.
