# Supplementary material for: Biomedical Text Link Prediction for Drug Discovery: A Case Study with COVID-19
Source: Pharmaceutics. 2021 May 26;13(6):794. doi: 10.3390/pharmaceutics13060794 (PMC8230210; doi:10.3390/pharmaceutics13060794)
Supplement: Supplementary file 1 [file pharmaceutics-13-00794-s001.zip › pharmaceutics-1195479-supplementary.pdf]

Table S1. *Top Ranked Link Prediction Nodes for Potential COVID-19 Treatment.* Predicted nodes are primarily based on analyzing connections in the knowledge graph from older coronavirus literature on SARS and MERS. Lower hetesim score is better. Standardize hetesim score used for comparison. Predicted link shows predicted relationship type. DOI, validation label, pharmacokinetic label determined by human in the loop validation using multiple trained human reviewers with quality control examining existing COVID-19 specific data sets.

node	Drug Class	node type	hetesim score	standardized hetesim score	novelty score	count score	predicted link 1	predicted link 2	predicted link 3	predicted link 4	DOI(s)	validation	pharmacokinetics
**Glycyrrhizic Acid**	anti-inflammatory	OrganicChemical	33.748	0.074	0.681	9.403	treats	prevents	part of	neg prevent	10.1016/j.phymed.2020.153364	PROVEN	Primary
**Quinine**	anti-inflammatory	OrganicChemical	35.013	0.077	0.665	9.297	treats	prevents	neg treats	neg prevent	10.1016/j.tmaid.2020.101873	PROVEN	Primary
**ebselen**	anti-inflammatory	OrganicChemical	35.015	0.077	0.679	9.583	treats	prevents	neg treats	inhibits	10.1016/j.freera	UNCLEAR	N/A
**Fluticasone propionate**	anti-inflammatory	OrganicChemical	36.184	0.079	0.670	9.603	treats	prevents	neg treats	part of	10.3390/v12040404	UNCLEAR	N/A
**Esters**	anti-inflammatory	OrganicChemical	32.110	0.070	0.650	8.510	N/A	N/A	N/A	N/A	N/A	MISSING	N/A
**Flavonoids**	anti-inflammatory	OrganicChemical	35.868	0.079	0.637	8.867	prevents	treats	part of	neg treats	10.1016/j.cbi.2020.109211	PROVEN	Primary
**Quinolone Antibacterial Agents**	anti-inflammatory	OrganicChemical	37.309	0.082	0.654	9.472	prevents	treats	part of	neg treats	10.3389/fmed.2020.00420	UNCLEAR	N/A
**Morphine**	anti-inflammatory	OrganicChemical	38.213	0.084	0.592	8.359	treats	prevents	neg treats	part of	10.1016/j.dsx.2020.05.022	UNCLEAR	N/A
**Curcumin**	anti-inflammatory	OrganicChemical	38.445	0.084	0.594	8.432	treats	prevents	part of	neg treats	10.1007/s13337-020-00598-8	UNCLEAR	N/A
**Hydroxymethylglutaryl-CoA Reductase Inhibitors**	anti-inflammatory	OrganicChemical	36.662	0.080	0.619	8.641	prevents	treats	neg treats	part of	10.1002/phar.2397	PROVEN	Side-Effect
**salicylate**	anti-inflammatory	OrganicChemical	38.471	0.084	0.643	9.441	treats	prevents	neg treats	part of	10.1080/22221751.2020.1785336	UNCLEAR	N/A
**baicalin**	anti-inflammatory	OrganicChemical	37.024	0.081	0.658	9.501	treats	prevents	part of	inhibits	10.1038/s41401-020-0483-6	PROVEN	Primary
**Albuterol**	anti-inflammatory	OrganicChemical	37.647	0.083	0.645	9.345	treats	prevents	neg treats	part of	10.1186/s10020-020-00215-w	PROVEN	Adjunctive
**Levamisole**	anti-inflammatory	OrganicChemical	38.954	0.085	0.624	9.143	prevents	treats	neg treats	part of	10.3855/jidc.13101	UNCLEAR	N/A
**ethyl pyruvate**	anti-inflammatory	OrganicChemical	36.503	0.080	0.679	9.829	treats	prevents	neg prevent	part of	N/A	MISSING	N/A
**Chlorpromazine**	anti-inflammatory	OrganicChemical	38.653	0.085	0.626	9.118	treats	prevents	part of	neg treats	10.1016/j.encep.2020.05.006	PROVEN	Primary
**Tretinoin**	anti-inflammatory	OrganicChemical	37.341	0.082	0.579	7.941	treats	prevents	neg treats	part of	N/A	MISSING	N/A
**Indomethacin**	anti-inflammatory	OrganicChemical	38.826	0.085	0.592	8.459	treats	prevents	part of	neg treats	10.1111/ijcp.13535	PROVEN	Adjunctive
**Sulfonamides**	anti-inflammatory	OrganicChemical	38.098	0.084	0.641	9.332	treats	prevents	neg treats	part of	N/A	MISSING	N/A
**SB 203580**	anti-inflammatory	OrganicChemical	38.160	0.084	0.653	9.599	treats	prevents	neg treats	part of	N/A	MISSING	N/A
**Chloroquine**	antimalarial	OrganicChemical	25.420	0.077	0.648	7.470	treats	prevents	neg treats	neg prevent	10.1016/j.virol.2020.08.011	PROVEN	Primary
**Fluoroquinolones**	antimalarial	OrganicChemical	25.554	0.077	0.682	8.125	treats	prevents	neg treats	part of	10.1016/j.ctim.2020.102473	PROVEN	Adjunctive
**Polyamines**	antimalarial	OrganicChemical	26.260	0.080	0.656	7.759	treats	prevents	part of	neg treats	10.1021/acsinfecdis.0c00491	DISPROVEN	Other
**Ciprofloxacin**	antimalarial	OrganicChemical	27.756	0.084	0.647	7.878	treats	prevents	neg treats	part of	10.1136/bmjgast-2020-000434	PROVEN	Side-Effect
**Tannic Acid**	antimalarial	OrganicChemical	28.577	0.087	0.666	8.391	treats	prevents	part of	neg treats	10.1111/jfbc.13432	UNCLEAR	N/A
**Vitamin A**	antimalarial	OrganicChemical	29.413	0.089	0.599	7.291	prevents	treats	neg treats	part of	10.18632/aging.103888	UNCLEAR	N/A

**Validation Label**
PROVEN
DISPROVEN
UNCLEAR
MISSING

**Pharmacokinetic Label**
Primary
Adjunctive
Side-Effect
Other

Table S1. *Top Ranked Link Prediction Nodes for Potential COVID-19 Treatment.* Predicted nodes are primarily based on analyzing connections in the knowledge graph from older coronavirus literature on SARS and MERS. Lower hetesim score is better. Standardize hetesim score used for comparison. Predicted link shows predicted relationship type. DOI, validation label, pharmacokinetic label determined by human in the loop validation using multiple trained human reviewers with quality control examining existing COVID-19 specific data sets.

node	Drug Class	node type	hetesim score	standardized hetesim score	novelty score	count score	predicted link 1	predicted link 2	predicted link 3	predicted link 4	DOI(s)	validation	pharmacokinetics
**Methotrexate**	antimalarial	OrganicChemical	28.656	0.087	0.602	7.211	treats	prevents	neg treats	part of	10.1002/jmv.26512	PROVEN	Primary
**Esters**	antimalarial	OrganicChemical	27.856	0.084	0.629	7.554	N/A	N/A	N/A	N/A	N/A	MISSING	N/A
**artemisinine**	antimalarial	OrganicChemical	28.721	0.087	0.666	8.416	treats	prevents	neg treats	part of	10.1021/acsinfecdis.0c00522	PROVEN	Primary
**Alkaloids**	antimalarial	OrganicChemical	29.719	0.090	0.652	8.342	prevents	treats	part of	neg treats	10.1007/s11101-020-09723-3	PROVEN	Primary
**Camptothecin**	antimalarial	OrganicChemical	28.953	0.088	0.669	8.510	treats	prevents	part of	neg treats	N/A	MISSING	N/A
**Aldehydes**	antimalarial	OrganicChemical	29.544	0.090	0.644	8.151	N/A	N/A	N/A	N/A	N/A	MISSING	N/A
**Vitamin K 3**	antimalarial	OrganicChemical	29.556	0.090	0.666	8.573	treats	prevents	part of	neg treats	N/A	MISSING	N/A
**Flavonoids**	antimalarial	OrganicChemical	29.755	0.090	0.630	7.937	prevents	treats	part of	neg treats	10.1016/j.cbi.2020.109211	PROVEN	Primary
**Amantadine**	antimalarial	OrganicChemical	30.196	0.092	0.655	8.487	prevents	treats	neg treats	neg prevent	10.1186/s40734-020-00089-4	UNCLEAR	N/A
**favor**	antimalarial	OrganicChemical	29.810	0.090	0.591	7.211	prevents	treats	part of	neg treats	N/A	MISSING	N/A
**Quinine**	antimalarial	OrganicChemical	30.175	0.091	0.648	8.351	treats	prevents	neg treats	neg prevent	10.1016/j.tmaid.2020.101873	PROVEN	Primary
**Quinolone Antibacterial Agents**	antimalarial	OrganicChemical	30.588	0.093	0.654	8.530	prevents	treats	part of	neg treats	10.3389/fmed.2020.00420	UNCLEAR	N/A
**quinoline**	antimalarial	OrganicChemical	31.022	0.094	0.667	8.855	part of	neg treats	treats	prevents	N/A	MISSING	N/A
**Sulfonamides**	antimalarial	OrganicChemical	31.433	0.095	0.634	8.328	treats	prevents	neg treats	part of	N/A	MISSING	N/A
**Antiviral Therapy**	antiviral agent	TherapeuticOrPreventiveProcedure	22.254	0.086	0.561	6.886	treats	neg treats	prevents	N/A	10.1136/thorax.	PROVEN	Primary
**prevention of disorder**	antiviral agent	TherapeuticOrPreventiveProcedure	23.438	0.091	0.614	8.266	N/A	N/A	N/A	N/A	N/A	MISSING	N/A
**Adoptive Transfer**	antiviral agent	TherapeuticOrPreventiveProcedure	24.425	0.095	0.620	8.624	prevents	treats	neg treats	N/A	N/A	MISSING	N/A
**Antiretroviral Therapy, Highly Active**	antiviral agent	TherapeuticOrPreventiveProcedure	25.461	0.099	0.606	8.605	neg treats	treats	prevents	N/A	https://pubmed.	UNCLEAR	N/A
**Influenza vaccination**	antiviral agent	TherapeuticOrPreventiveProcedure	25.746	0.100	0.606	8.674	prevents	treats	neg treats	N/A	N/A	MISSING	N/A
**Clinical Treatment**	antiviral agent	TherapeuticOrPreventiveProcedure	25.859	0.100	0.606	8.711	N/A	N/A	N/A	N/A	N/A	MISSING	N/A
**Therapeutic immunosuppression**	antiviral agent	TherapeuticOrPreventiveProcedure	26.364	0.102	0.539	7.470	prevents	neg treats	treats	N/A	https://doi.org/1	UNCLEAR	N/A
**Hematopoietic Stem Cell Transplantation**	antiviral agent	TherapeuticOrPreventiveProcedure	26.404	0.102	0.581	8.334	treats	neg treats	prevents	N/A	DOI: 10.1038/s41392-020-00286-5	UNCLEAR	N/A
**Vaccination**	antiviral agent	TherapeuticOrPreventiveProcedure	26.233	0.102	0.405	4.697	treats	prevents	neg treats	N/A	DOI: 10.1016/S0	PROVEN	Primary
**Empirical therapy**	antiviral agent	TherapeuticOrPreventiveProcedure	26.556	0.103	0.614	9.051	treats	prevents	neg treats	N/A	N/A	MISSING	N/A

Table S1. *Top Ranked Link Prediction Nodes for Potential COVID-19 Treatment.* Predicted nodes are primarily based on analyzing connections in the knowledge graph from older coronavirus literature on SARS and MERS. Lower hetesim score is better. Standardize hetesim score used for comparison. Predicted link shows predicted relationship type. DOI, validation label, pharmacokinetic label determined by human in the loop validation using multiple trained human reviewers with quality control examining existing COVID-19 specific data sets.

node	Drug Class	node type	hetesim score	standardized hetesim score	novelty score	count score	predicted link 1	predicted link 2	predicted link 3	predicted link 4	DOI(s)	validation	pharmaco kinetics
**HIV therapy**	antiviral agent	TherapeuticOrPreventiveProcedure	26.527	0.103	0.635	9.469	neg treats	treats	prevents	N/A	DOI: 10.1586/14	UNCLEAR	N/A
**early identification**	antiviral agent	TherapeuticOrPreventiveProcedure	26.904	0.104	0.583	8.498	treats	prevents	neg treats	N/A	N/A	MISSING	N/A
**interferon therapy**	antiviral agent	TherapeuticOrPreventiveProcedure	27.117	0.105	0.615	9.221	prevents	treats	neg treats	N/A	DOI: 10.1517/14	PROVEN	Primary
**Immunotherapy**	antiviral agent	TherapeuticOrPreventiveProcedure	27.345	0.106	0.488	6.677	treats	prevents	neg treats	N/A	DOI: 10.1016/j.ir	UNCLEAR	N/A
**Cytomegalovirus prophylaxis**	antiviral agent	TherapeuticOrPreventiveProcedure	27.210	0.105	0.645	9.847	N/A	N/A	N/A	N/A	N/A	MISSING	N/A
**Post-exposure prophylaxis**	antiviral agent	TherapeuticOrPreventiveProcedure	27.420	0.106	0.607	9.123	prevents	treats	neg treats	N/A	N/A	MISSING	N/A
**The science and art of healing**	antiviral agent	TherapeuticOrPreventiveProcedure	27.920	0.108	0.544	7.954	N/A	N/A	N/A	N/A	N/A	MISSING	N/A
**Therapeutic Intervention**	antiviral agent	TherapeuticOrPreventiveProcedure	28.210	0.109	0.509	7.308	treats	neg treats	prevents	N/A	N/A	MISSING	N/A
**Biological Response Modifier Therapy**	antiviral agent	TherapeuticOrPreventiveProcedure	28.245	0.109	0.591	9.000	treats	prevents	neg treats	N/A	N/A	MISSING	N/A
**Bone Marrow Transplantation**	antiviral agent	TherapeuticOrPreventiveProcedure	27.609	0.107	0.549	7.976	treats	prevents	neg treats	N/A	doi: 10.1089/scd	PROVEN	Adjunctive
**Capsid Proteins**	envelope protein	AminoAcidPeptideOrProtein	6.355	0.079	0.612	6.742	part of	neg part of	treats	prevents	10.1016/j.anndia	PROVEN	Other
**Viral Proteins**	envelope protein	AminoAcidPeptideOrProtein	6.266	0.078	0.599	6.399	neg part of	part of	treats	prevents	N/A	MISSING	N/A
**Flagellin**	envelope protein	AminoAcidPeptideOrProtein	7.146	0.089	0.688	8.931	treats	neg part of	part of	prevents	10.1016/j.ebiom.2020.102743	UNCLEAR	N/A
**Gene Products, env**	envelope protein	AminoAcidPeptideOrProtein	6.994	0.087	0.673	8.493	neg part of	part of	treats	compared w	N/A	MISSING	N/A
**Structural protein**	envelope protein	AminoAcidPeptideOrProtein	7.312	0.091	0.627	7.719	N/A	N/A	N/A	N/A	N/A	MISSING	N/A
**Neuraminidase**	envelope protein	AminoAcidPeptideOrProtein	7.707	0.096	0.634	8.144	neg part of	treats	part of	prevents	10.1097/JCMA.0000000000000353	PROVEN	Other
**Nucleocapsid**	envelope protein	AminoAcidPeptideOrProtein	7.731	0.097	0.642	8.322	part of	neg part of	compared w	treats	10.1016/j.jmb.2(	PROVEN	Other
**Chemokine (C-C Motif) Receptor 5|CCR5**	envelope protein	Receptor	8.312	0.104	0.652	8.941	treats	neg part of	part of	prevents	10.1016/j.antivir	DISPROVEN	Other
**Viral Core Proteins**	envelope protein	AminoAcidPeptideOrProtein	8.154	0.102	0.634	8.436	neg part of	part of	treats	prevents	N/A	MISSING	N/A
**Polymerase|ERVK-9|ERVK-19|ERVK-11**	envelope protein	AminoAcidPeptideOrProtein	8.238	0.103	0.603	7.826	N/A	N/A	N/A	N/A	N/A	MISSING	N/A

Table S1. *Top Ranked Link Prediction Nodes for Potential COVID-19 Treatment.* Predicted nodes are primarily based on analyzing connections in the knowledge graph from older coronavirus literature on SARS and MERS. Lower hetesim score is better. Standardize hetesim score used for comparison. Predicted link shows predicted relationship type. DOI, validation label, pharmacokinetic label determined by human in the loop validation using multiple trained human reviewers with quality control examining existing COVID-19 specific data sets.

node	Drug Class	node type	hetesim score	standardized hetesim score	novelty score	count score	predicted link 1	predicted link 2	predicted link 3	predicted link 4	DOI(s)	validation	pharmaco kinetics
**Gene Products, gag**	envelope protein	AminoAcidPeptideOrProtein	8.373	0.105	0.650	8.938	part of	neg part of	treats	compared w	N/A	MISSING	N/A
**Virus-neutralizing Antibody**	envelope protein	ImmunologicFactor	8.491	0.106	0.650	9.017	prevents	part of	treats	neg part of	10.3390/vaccines8030404	UNCLEAR	N/A
**Membrane Fusion Proteins**	envelope protein	AminoAcidPeptideOrProtein	9.376	0.117	0.531	7.040	part of	neg part of	treats	neg treats	10.1016/j.antivi	DISPROVEN	Other
**synthetic peptide**	envelope protein	AminoAcidPeptideOrProtein	8.768	0.110	0.591	7.925	part of	treats	prevents	neg part of	10.1016/j.meegid.2020.104474	UNCLEAR	N/A
**Interferon Alpha, Human|IFNA1**	envelope protein	ImmunologicFactor	8.770	0.110	0.625	8.655	N/A	N/A	N/A	N/A	N/A	MISSING	N/A
**Bacterial Proteins**	envelope protein	AminoAcidPeptideOrProtein	9.488	0.119	0.634	9.353	part of	treats	neg part of	prevents	N/A	MISSING	N/A
**HIV Envelope Protein gp160 (gp160|ENPEP)**	envelope protein	AminoAcidPeptideOrProtein	9.188	0.115	0.647	9.425	part of	neg part of	treats	prevents	10.1128/JVI.78.19.10328-10335.2004	UNCLEAR	N/A
**Polyclonal antibody**	envelope protein	ImmunologicFactor	9.271	0.116	0.578	7.988	treats	part of	prevents	neg part of	10.1007/s10930-020-09921-0	UNCLEAR	N/A
**Receptors, Virus**	envelope protein	Receptor	8.788	0.110	0.688	10.050	part of	neg part of	prevents	treats	10.1007/s10561-020-09869-6	PROVEN	Other
**Ubiquitin**	envelope protein	AminoAcidPeptideOrProtein	9.449	0.118	0.559	7.695	part of	neg part of	treats	prevents	10.3389/fmolb.2020.00174	UNCLEAR	N/A
**small molecule**	glycoproteins big	OrganicChemical	31.411	0.056	0.733	9.705	prevents	treats	part of	neg treats	N/A	MISSING	N/A
**Chloroquine**	glycoproteins big	OrganicChemical	40.646	0.073	0.709	10.306	treats	prevents	neg treats	neg prevent	10.1016/j.virol.2020.08.011	PROVEN	Primary
**RABBIT SERUM**	glycoproteins big	OrganicChemical	33.844	0.061	0.756	10.575	N/A	N/A	N/A	N/A	N/A	MISSING	N/A
**Edetic Acid**	glycoproteins big	OrganicChemical	40.971	0.074	0.701	10.141	treats	prevents	neg treats	part of	N/A	MISSING	N/A
**Biotin**	glycoproteins big	OrganicChemical	42.352	0.076	0.695	10.169	prevents	part of	treats	N/A	N/A	MISSING	N/A
**Fluoroquinolones**	glycoproteins big	OrganicChemical	46.814	0.084	0.716	11.245	treats	prevents	neg treats	part of	10.1016/j.ctim.2020.102473	PROVEN	Adjunctive
**Methotrexate**	glycoproteins big	OrganicChemical	48.234	0.087	0.658	10.030	treats	prevents	neg treats	part of	DOI: 10.1111/dtl	UNCLEAR	N/A
**Vitamin A**	glycoproteins big	OrganicChemical	48.329	0.087	0.667	10.248	prevents	treats	neg treats	part of	10.18632/aging.103888	UNCLEAR	N/A
**Sulfhydryl Compounds**	glycoproteins big	OrganicChemical	45.896	0.083	0.665	9.889	treats	prevents	part of	neg treats	N/A	MISSING	N/A
**Pentetic Acid**	glycoproteins big	OrganicChemical	46.035	0.083	0.723	11.328	treats	prevents	neg treats	part of	N/A	MISSING	N/A
**Esters**	glycoproteins big	OrganicChemical	46.060	0.083	0.678	10.240	N/A	N/A	N/A	N/A	N/A	MISSING	N/A
**Adriamycin**	glycoproteins big	OrganicChemical	49.257	0.089	0.680	10.678	treats	prevents	part of	neg treats	10.1016/j.compb	UNCLEAR	N/A
**Ethylene**	glycoproteins big	OrganicChemical	46.668	0.084	0.714	11.183	treats	prevents	part of	neg treats	N/A	MISSING	N/A
**2,5-dichloro-4-bromophenol**	glycoproteins big	OrganicChemical	46.752	0.084	0.726	11.501	prevents	part of	treats	neg prevent	N/A	MISSING	N/A
**Daunorubicin**	glycoproteins big	OrganicChemical	47.436	0.085	0.707	11.125	treats	part of	neg treats	interacts wit	N/A	MISSING	N/A
**favor**	glycoproteins big	OrganicChemical	48.312	0.087	0.660	10.085	prevents	treats	part of	neg treats	N/A	MISSING	N/A
**poly(2-acrylamido-2-methyl-1-propanesulfonic acid)**	glycoproteins big	OrganicChemical	48.656	0.088	0.718	11.548	N/A	N/A	N/A	N/A	N/A	MISSING	N/A
**fucoidan**	glycoproteins big	OrganicChemical	48.601	0.087	0.709	11.301	treats	prevents	neg prevent:	neg treats	DOI: 10.1039/d0	PROVEN	Primary
**Tretinoin**	glycoproteins big	OrganicChemical	49.687	0.089	0.635	9.633	treats	prevents	neg treats	part of	N/A	MISSING	N/A
**Cytembena**	glycoproteins big	OrganicChemical	50.128	0.090	0.710	11.533	N/A	N/A	N/A	N/A	N/A	MISSING	N/A
**small molecule**	immunomodulators	OrganicChemical	39.124	0.069	0.662	8.825	prevents	treats	part of	neg treats	https://pubmed.	UNCLEAR	N/A
**Chloroquine**	immunomodulators	OrganicChemical	47.007	0.083	0.626	9.114	treats	prevents	neg treats	neg prevent	https://www.ncl	PROVEN	Primary
**Fluoroquinolones**	immunomodulators	OrganicChemical	44.706	0.079	0.674	9.840	treats	prevents	neg treats	part of	N/A	MISSING	N/A
**Ciprofloxacin**	immunomodulators	OrganicChemical	48.003	0.085	0.641	9.581	treats	prevents	neg treats	part of	10.1136/bmjgast-2020-000434	PROVEN	Side-Effect

Table S1. *Top Ranked Link Prediction Nodes for Potential COVID-19 Treatment.* Predicted nodes are primarily based on analyzing connections in the knowledge graph from older coronavirus literature on SARS and MERS. Lower hetesim score is better. Standardize hetesim score used for comparison. Predicted link shows predicted relationship type. DOI, validation label, pharmacokinetic label determined by human in the loop validation using multiple trained human reviewers with quality control examining existing COVID-19 specific data sets.

node	Drug Class	node type	hetesim score	standardized hetesim score	novelty score	count score	predicted link 1	predicted link 2	predicted link 3	predicted link 4	DOI(s)	validation	pharmaco kinetics
**fucoidan**	immunomodulators	OrganicChemical	46.633	0.082	0.670	10.024	treats	prevents	neg prevent	neg treats	N/A	MISSING	N/A
**Biotin**	immunomodulators	OrganicChemical	49.327	0.087	0.612	9.117	prevents	part of	treats	neg treats	https://www.ncl	UNCLEAR	N/A
**Vitamin A**	immunomodulators	OrganicChemical	50.576	0.089	0.598	8.984	prevents	treats	neg treats	part of	https://www.ncl	UNCLEAR	N/A
**artemisinine**	immunomodulators	OrganicChemical	49.243	0.087	0.656	10.078	treats	prevents	neg treats	part of	10.1021/acsinfecdis.0c00522	PROVEN	Primary
**Flavonoids**	immunomodulators	OrganicChemical	50.729	0.090	0.625	9.585	prevents	treats	part of	neg treats	https://www.ncl	UNCLEAR	N/A
**docetaxel**	immunomodulators	OrganicChemical	49.562	0.088	0.649	9.968	treats	prevents	part of	neg prevent	N/A	MISSING	N/A
**Nelfinavir**	immunomodulators	OrganicChemical	51.126	0.090	0.657	10.342	treats	prevents	neg treats	part of	10.3390/v12101178	UNCLEAR	N/A
**temozolomide**	immunomodulators	OrganicChemical	51.265	0.091	0.651	10.240	treats	prevents	neg prevent	part of	https://www.ncl	UNCLEAR	N/A
**polyphenols**	immunomodulators	OrganicChemical	51.447	0.091	0.634	9.891	treats	prevents	part of	neg treats	https://pubmed.ncbi.nlm.nih.	UNCLEAR	N/A
**Ginseng Preparation**	immunomodulators	OrganicChemical	51.939	0.092	0.650	10.293	prevents	treats	part of	neg treats	https://pubmed.	UNCLEAR	N/A
**Tretinoin**	immunomodulators	OrganicChemical	50.727	0.090	0.575	8.511	treats	prevents	neg treats	part of	N/A	MISSING	N/A
**sphingosine 1-phosphate**	immunomodulators	OrganicChemical	51.373	0.091	0.634	9.873	prevents	treats	neg treats	part of	N/A	MISSING	N/A
**Paclitaxel**	immunomodulators	OrganicChemical	51.438	0.091	0.611	9.382	treats	prevents	neg treats	part of	N/A	MISSING	N/A
**Hydroxymethylglutaryl-CoA Reductase Inhibitors**	immunomodulators	OrganicChemical	51.537	0.091	0.610	9.371	prevents	treats	neg treats	part of	N/A	MISSING	N/A
**Mistletoe preparation**	immunomodulators	OrganicChemical	51.853	0.092	0.660	10.506	N/A	N/A	N/A	N/A	N/A	MISSING	N/A
**bryostatin 1**	immunomodulators	OrganicChemical	52.082	0.092	0.657	10.463	treats	prevents	neg prevent	neg treats	N/A	MISSING	N/A
**Neuraminidase**	neuraminidase inhibitors	AminoAcidPeptideOrProtein	6.300	0.085	0.624	4.738	neg part of	treats	part of	prevents	N/A	MISSING	N/A
**Complement System Proteins**	neuraminidase inhibitors	ImmunologicFactor	7.469	0.101	0.549	4.595	prevents	part of	treats	neg part of	https://pubmed.	DISPROVEN	Other
**Dopamine Receptor**	neuraminidase inhibitors	Receptor	7.603	0.103	0.580	4.990	treats	neg part of	part of	prevents	https://pubmed.	DISPROVEN	Other
**human leukocyte interferon|IFNA1**	neuraminidase inhibitors	ImmunologicFactor	7.032	0.095	0.583	4.716	treats	prevents	neg part of	part of	https://pubmed.	PROVEN	Primary
**Sialyltransferases**	neuraminidase inhibitors	AminoAcidPeptideOrProtein	7.243	0.098	0.603	5.036	part of	neg part of	treats	administere	N/A	UNCLEAR	N/A
**Glycoside Hydrolases**	neuraminidase inhibitors	AminoAcidPeptideOrProtein	7.302	0.099	0.598	5.010	treats	part of	neg treats	neg part of	https://pubmed.	UNCLEAR	Primary
**neuraminidases**	neuraminidase inhibitors	AminoAcidPeptideOrProtein	7.152	0.097	0.605	5.001	part of	neg part of	treats	prevents	N/A	MISSING	N/A
**Polymerase|ERVK-9|ERVK-19|ERVK-11**	neuraminidase inhibitors	AminoAcidPeptideOrProtein	7.178	0.097	0.598	4.944	N/A	N/A	N/A	N/A	N/A	MISSING	N/A
**Polymerase**	neuraminidase inhibitors	AminoAcidPeptideOrProtein	7.191	0.097	0.602	4.994	part of	neg part of	treats	compared w	N/A	MISSING	N/A
**Ion Channel**	neuraminidase inhibitors	AminoAcidPeptideOrProtein	7.272	0.098	0.591	4.921	part of	neg part of	prevents	treats	N/A	MISSING	N/A
**Brain-Derived Neurotrophic Factor**	neuraminidase inhibitors	AminoAcidPeptideOrProtein	7.536	0.102	0.570	4.851	part of	treats	prevents	stimulates	https://pubmed.	UNCLEAR	N/A
**PER2 protein, mammalian**	neuraminidase inhibitors	AminoAcidPeptideOrProtein	7.437	0.100	0.585	4.953	neg part of	part of	treats	stimulates	N/A	MISSING	N/A
**Mannose Binding Lectin**	neuraminidase inhibitors	AminoAcidPeptideOrProtein	7.321	0.099	0.582	4.864	treats	part of	prevents	neg part of	https://pubmed.	PROVEN	Primary
**Recombinant Interferon-gamma**	neuraminidase inhibitors	ImmunologicFactor	7.534	0.102	0.558	4.734	treats	prevents	part of	neg part of	https://pubmed.	PROVEN	Primary
**Cyclosporine**	neuraminidase inhibitors	AminoAcidPeptideOrProtein	7.658	0.103	0.553	4.746	treats	prevents	neg part of	part of	https://pubmed.	PROVEN	Primary

Table S1. *Top Ranked Link Prediction Nodes for Potential COVID-19 Treatment.* Predicted nodes are primarily based on analyzing connections in the knowledge graph from older coronavirus literature on SARS and MERS. Lower hetesim score is better. Standardize hetesim score used for comparison. Predicted link shows predicted relationship type. DOI, validation label, pharmacokinetic label determined by human in the loop validation using multiple trained human reviewers with quality control examining existing COVID-19 specific data sets.

node	Drug Class	node type	hetesim score	standardized hetesim score	novelty score	count score	predicted link 1	predicted link 2	predicted link 3	predicted link 4	DOI(s)	validation	pharmacokinetics
**Nerve Growth Factors**	neuraminidase inhibitors	AminoAcidPeptideOrProtein	7.647	0.103	0.560	4.807	treats	part of	prevents	neg part of	https://pubmed.	UNCLEAR	N/A
**Ion Channel Protein**	neuraminidase inhibitors	AminoAcidPeptideOrProtein	7.805	0.105	0.580	5.100	part of	neg part of	compared w	treats	N/A	MISSING	N/A
**Amino Acids**	neuraminidase inhibitors	AminoAcidPeptideOrProtein	7.873	0.106	0.524	4.567	treats	prevents	part of	neg part of	N/A	MISSING	N/A
**Azaserine**	neuraminidase inhibitors	AminoAcidPeptideOrProtein	8.013	0.108	0.569	5.107	treats	neg part of	part of	neg prevent	N/A	MISSING	N/A
**sialic acid receptor**	neuraminidase inhibitors	Receptor	7.914	0.107	0.571	5.076	neg part of	part of	prevents	compared w	https://pubmed.	PROVEN	Other
**Antiviral Therapy**	nucleoside analogs	TherapeuticOrPreventiveProcedure	14.801	0.093	0.572	10.781	treats	neg treats	prevents	neg prevent	DOI: 10.1080/17	PROVEN	Primary
**Antiretroviral Therapy, Highly Active**	nucleoside analogs	TherapeuticOrPreventiveProcedure	14.942	0.093	0.565	10.707	neg treats	treats	prevents	neg prevent	N/A	MISSING	N/A
**Nucleoside Analogs**	nucleoside analogs	PharmacologicSubstance	15.776	0.099	0.554	10.897	treats	neg treats	prevents	part OF	N/A	UNCLEAR	N/A
**Therapeutic immunosuppression**	nucleoside analogs	TherapeuticOrPreventiveProcedure	16.262	0.102	0.522	10.369	prevents	neg treats	treats	neg prevent	https://doi.org/1	UNCLEAR	N/A
**interferon therapy**	nucleoside analogs	TherapeuticOrPreventiveProcedure	15.689	0.098	0.569	11.250	prevents	treats	neg treats	neg prevent	DOI: 10.1517/14	PROVEN	Primary
**Antiviral prophylaxis**	nucleoside analogs	TherapeuticOrPreventiveProcedure	17.182	0.107	0.577	12.352	neg treats	prevents	treats	neg prevent	DOI: 10.1080/17	DISPROVEN	Primary
**gene therapy**	nucleoside analogs	TherapeuticOrPreventiveProcedure	16.409	0.103	0.515	10.258	treats	neg treats	prevents	neg prevent	DOI: 10.3390/bi	UNCLEAR	N/A
**Chemotherapy-Oncologic Procedure**	nucleoside analogs	TherapeuticOrPreventiveProcedure	16.319	0.102	0.499	9.809	N/A	N/A	N/A	N/A	N/A	MISSING	N/A
**Combination Drug Therapy**	nucleoside analogs	TherapeuticOrPreventiveProcedure	16.492	0.103	0.524	10.564	treats	prevents	neg treats	neg prevent	DOI: 10.1371/jou	MISSING	N/A
**HIV therapy**	nucleoside analogs	TherapeuticOrPreventiveProcedure	16.946	0.106	0.584	12.405	neg treats	treats	prevents	neg prevent	DOI: 10.1586/14	UNCLEAR	N/A
**Cytotoxic Chemotherapy**	nucleoside analogs	TherapeuticOrPreventiveProcedure	16.513	0.103	0.556	11.396	treats	prevents	neg treats	neg prevent	N/A	MISSING	N/A
**Maintenance therapy**	nucleoside analogs	TherapeuticOrPreventiveProcedure	16.607	0.104	0.519	10.497	treats	prevents	neg treats	neg prevent	N/A	MISSING	N/A
**Lamivudine**	nucleoside analogs	PharmacologicSubstance	16.726	0.105	0.493	9.889	treats	prevents	neg treats	part OF	N/A	MISSING	N/A
**Salvage Therapy**	nucleoside analogs	TherapeuticOrPreventiveProcedure	16.751	0.105	0.540	11.122	treats	neg treats	prevents	neg prevent	N/A	MISSING	N/A

Table S1. *Top Ranked Link Prediction Nodes for Potential COVID-19 Treatment.* Predicted nodes are primarily based on analyzing connections in the knowledge graph from older coronavirus literature on SARS and MERS. Lower hetesim score is better. Standardize hetesim score used for comparison. Predicted link shows predicted relationship type. DOI, validation label, pharmacokinetic label determined by human in the loop validation using multiple trained human reviewers with quality control examining existing COVID-19 specific data sets.

node	Drug Class	node type	hetesim score	standardized hetesim score	novelty score	count score	predicted link 1	predicted link 2	predicted link 3	predicted link 4	DOI(s)	validation	pharmaco kinetics
**Psychotherapy, Multiple**	nucleoside analogs	TherapeuticOrPre<br>ventiveProcedure	16.825	0.105	0.506	10.275	treats	prevents	neg treats	neg prevent	N/A	MISSING	N/A
**Clinical Treatment**	nucleoside analogs	TherapeuticOrPre<br>ventiveProcedure	16.990	0.106	0.544	11.390	N/A	N/A	N/A	N/A	N/A	MISSING	N/A
**Zidovudine**	nucleoside analogs	PharmacologicSu<br>bstance	16.881	0.106	0.474	9.482	neg treats	treats	prevents	part OF	DOI: 10.1016/j.a	PROVEN	Primary
**Cancer Treatment**	nucleoside analogs	TherapeuticOrPre<br>ventiveProcedure	17.125	0.107	0.499	10.270	N/A	N/A	N/A	N/A	N/A	MISSING	N/A
**Retreatments**	nucleoside analogs	TherapeuticOrPre<br>ventiveProcedure	17.172	0.107	0.539	11.357	treats	neg treats	prevents	neg prevent	N/A	MISSING	N/A
**Plasmapheresis**	nucleoside analogs	TherapeuticOrPre<br>ventiveProcedure	17.115	0.107	0.554	11.709	prevents	neg treats	treats	neg prevent	DOI: 10.1016/j.b	UNCLEAR	N/A
**small molecule**	protease inhibitors	OrganicChemical	28.070	0.075	0.622	8.770	N/A	N/A	N/A	N/A	N/A	MISSING	N/A
**Chloroquine**	protease inhibitors	OrganicChemical	31.185	0.084	0.613	9.249	treats	prevents	neg treats	neg prevent	10.1016/j.virol.<br>2020.08.011	PROVEN	Primary
**Edetic Acid**	protease inhibitors	OrganicChemical	31.726	0.085	0.604	9.158	treats	prevents	neg treats	part of	N/A	MISSING	N/A
**Ethylene**	protease inhibitors	OrganicChemical	33.955	0.091	0.629	10.201	treats	prevents	part of	neg treats	N/A	MISSING	N/A
**Pentetic Acid**	protease inhibitors	OrganicChemical	33.993	0.091	0.633	10.298	treats	prevents	neg treats	part of	N/A	MISSING	N/A
**Sulfhydryl Compounds**	protease inhibitors	OrganicChemical	34.020	0.091	0.574	8.973	treats	prevents	part of	neg treats	N/A	MISSING	N/A
**Vitamin A**	protease inhibitors	OrganicChemical	34.314	0.092	0.582	9.231	prevents	treats	neg treats	part of	10.18632/aging<br>.103888	UNCLEAR	N/A
**Esters**	protease inhibitors	OrganicChemical	34.115	0.092	0.586	9.272	N/A	N/A	N/A	N/A	N/A	MISSING	N/A
**Adriamycin**	protease inhibitors	OrganicChemical	34.633	0.093	0.598	9.649	treats	prevents	part of	neg treats	10.1016/j.compl	UNCLEAR	N/A
**favor**	protease inhibitors	OrganicChemical	34.893	0.094	0.575	9.184	prevents	treats	part of	neg treats	N/A	MISSING	N/A
**Amprenavir**	protease inhibitors	OrganicChemical	35.924	0.097	0.632	10.685	treats	prevents	part of	neg treats	10.1128/mSph<br>ere.00658-20	PROVEN	Primary
**Nelfinavir**	protease inhibitors	OrganicChemical	36.656	0.099	0.620	10.583	treats	prevents	neg treats	part of	10.1128/mBio.0	UNCLEAR	N/A
**Dimethyl Sulfoxide**	protease inhibitors	OrganicChemical	35.013	0.094	0.586	9.459	treats	prevents	neg treats	part of	N/A	MISSING	N/A
**Salicylic Acid**	protease inhibitors	OrganicChemical	36.658	0.099	0.601	10.140	prevents	treats	part of	neg treats	10.1016/j.jaccas	UNCLEAR	N/A
**Tretinoin**	protease inhibitors	OrganicChemical	35.355	0.095	0.549	8.702	treats	prevents	neg treats	part of	N/A	MISSING	N/A
**Curcumin**	protease inhibitors	OrganicChemical	36.873	0.099	0.561	9.311	treats	prevents	part of	neg treats	10.1007/s1333<br>7-020-00598-8	UNCLEAR	N/A
**Paclitaxel**	protease inhibitors	OrganicChemical	35.554	0.096	0.588	9.615	treats	prevents	neg treats	part of	N/A	MISSING	N/A
**2-Mercaptoethanol**	protease inhibitors	OrganicChemical	35.756	0.096	0.595	9.815	treats	part of	neg treats	prevents	N/A	MISSING	N/A
**Suramin**	protease inhibitors	OrganicChemical	36.563	0.098	0.599	10.082	prevents	treats	neg treats	part of	10.1128/AAC.0<br>0900-20	PROVEN	Primary
**Hydroxymethylglutaryl-CoA Reductase Inhibitors**	protease inhibitors	OrganicChemical	36.703	0.099	0.578	9.650	prevents	treats	neg treats	part of	10.1002/phar.2<br>397	PROVEN	Side-Effect
